# Emergence of a mortality disparity between a marginal rural area and the rest of Denmark, 1968-2017

**DOI:** 10.1186/s12889-020-10108-6

**Published:** 2021-01-07

**Authors:** Therese L. F. Holmager, Lars Thygesen, Lene T. Buur, Elsebeth Lynge

**Affiliations:** 1 Centre for Epidemiological Research, Nykøbing Falster Hospital, University of Copenhagen, Ejegodvej 63, DK-4800 Nykøbing Falster, Copenhagen, Denmark; 2 Thygesen Statistics Consulting, Copenhagen, Denmark; 3 Museum Lolland-Falster, Frisegade 40, DK-4800 Nykøbing Falster, Copenhagen, Denmark

**Keywords:** Denmark, Vital statistics, Health status disparities, Population dynamics, Rural population

## Abstract

**Background:**

Lolland-Falster is a rural area of Denmark, where the life expectancy is presently almost six years lower than in the rich capital suburbs. To determine the origin of this disparity, we analysed changes in mortality during 50 years in Lolland-Falster.

**Methods:**

Annual population number and number of deaths at municipality level were retrieved from StatBank Denmark and from Statistics Denmark publications, 1968–2017. For 1974–2017, life expectancy at birth by sex and 5-year calendar period was calculated. From 1968 to 2017, standardised mortality ratio (SMR) for all-cause mortality was calculated by sex, 5-year calendar period and municipality, with Denmark as standard and including 95% confidence intervals (CI).

**Results:**

In 1968–2017, life expectancy in Lolland-Falster increased, but less so than in the rest of Denmark. Fifty years ago, Lolland-Falster had a mortality similar to the rest of Denmark. The increasing mortality disparity developed gradually starting in the late 1980s, earlier in Lolland municipality (western part) than in Guldborgsund municipality (eastern part), and earlier for men than for women. By 2013–2017, the SMR had reached 1.25 (95% CI 1.19–1.31) for men in the western part, and 1.11 (95% CI 1.08–1.16) for women in the eastern part. Increasing mortality disparity was particularly seen in people aged 20–69 years.

**Conclusions:**

This study is the first to report on increasing geographical segregation in all-cause mortality in a Nordic welfare state. Development of the mortality disparity between Lolland-Falster and the rest of Denmark followed changes in agriculture, industrial company closure, a shipyard close-down, administrative centralisation, and a decreasing population size.

**Supplementary Information:**

The online version contains supplementary material available at 10.1186/s12889-020-10108-6.

## Background

Life expectancy is the most comprehensive measure of the health status in a population. For the larger part of time since the end of World War II, a general increase has been seen in life expectancy in Western, high-income countries [1]. However, this tendency has changed recently. In the European Union, the flu epidemic of 2014–2015 caused a temporary decrease in life expectancy, whereafter a stagnation has been observed [2]. In the US, the increase in life expectancy stopped in 2010, and it decreased slightly from 2014 to 2017 [3].

The decreased life expectancy in the US followed a widening gap in the life expectancy across states. In 1984, the maximum difference between states was 4.9 years; a span that had increased to 7.0 years by 2016 [3]. Urban US has improved its mortality faster than rural US [4]. In particular the development in mortality in the rural parts of Arkansas, Louisiana, Oklahoma, and Texas (classified as West South Central) and the rural parts of Alabama, Kentucky, Mississippi, and Tennessee (classified as East South Central) lagged behind that of the rest of the US [4]. The US decrease in life expectancy has been driven primarily by an increase in the mortality of people aged 25–44 years, and to a lesser extent by people aged 45–64 years [3]. Especially deaths from drug overdose, alcohol, suicide, organ system disease, and injuries have caused increase in mortality [3].

The increase and widening inequality in midlife mortality in the US started in a period marked by a major transformation in the nation’s economy, substantial job losses in manufacturing and other sectors, contraction of the middle class, wage stagnation, and reduced intergenerational mobility [3]. The question arises whether similar trends in mortality can be under way in other high-income countries with similar macroeconomic changes.

Another example of relative changes in all-cause mortality comes from Japan. Here Okinawa was known as the prefecture with the highest life expectancy, but this position changed over time [5]. While men in Okinawa had an about 8% mortality deficit in 1990 as compared with the average of Japanese men; equal mortality levels were seen in 2000. This relative change has been hypothesized to be related to a tendency among younger residents of Okinawa to avoid the traditional diet [5].

Up until 1980, the Nordic welfare states, Denmark, Norway and Sweden, were world-leaders in life expectancy, but around 1980 a stagnation occurred in Denmark, a phenomenon that lasted until the late 1990s [6, 7]. The stagnation in Danish life expectancy was largely attributable to tobacco smoking [7, 8]. Even if Denmark during the last years has experienced a faster increase in life expectancy than the best performing countries [7], Denmark is still positioned as only number 21 out of the 39 European countries [9]. In 2017–2018, the Danish life expectancy was 81.0 years [10].

Denmark has slightly less than 6 million inhabitants [11]. The country is normally considered to be homogenous and among the happiest countries in the world [11, 12]. This is in some way in contradiction to being only number 21 of European countries in life expectancy, when taking the WHO definition of health into account, where “Health is a state of complete physical, mental and social well-being and not merely the absence of disease or infirmity.” [13].

It is at the same time surprising that in 2013–2017, Denmark actually had large geographical disparities in life expectancy from 77.4 years in the south-eastern, rural Lolland-Falster to 83.1 in the rich suburbs north of the Copenhagen capital [14]. This is a difference of 5.7 years, equivalent to the change over a 20–30 years period at the national level [14]. Mortality-wise, the people of Lolland-Falster are thus a generation behind the people of the rich capital suburbs [14].

The presently low life expectancy in Lolland-Falster raises the question on whether Denmark might be on the same path towards increased geographical inequality in mortality as seen in the US.

On this background, we analysed the mortality of the inhabitants in Lolland-Falster during the past 50 years, both for the total population and by main age groups, and we compared the situation in Lolland-Falster with that in all of Denmark. The purpose of this paper is to investigate whether a high mortality is an inherent part of life in Lolland-Falster, or whether it is a new phenomenon.

## Methods

### Data sources

**Fig. 1:**
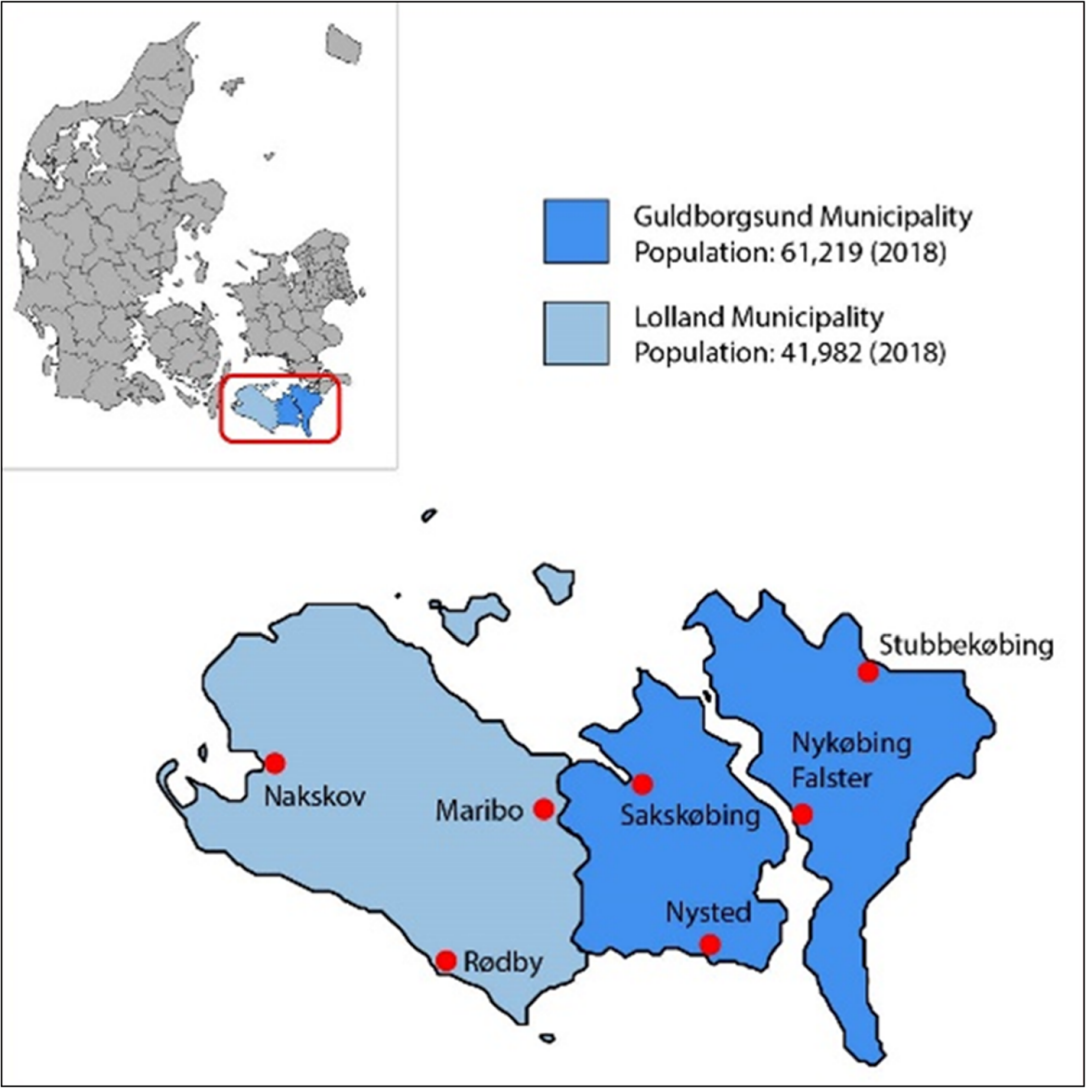
Map of Denmark with Lolland and Guldborgsund municipalities. The figure was made using open access templates from GeoDanmark, Available from: https://download.kortforsyningen.dk/content/geodanmark. Note: In 1968–1969, Lolland municipality was identified as the provincial cities; Maribo, Nakskov and Rødby, and the parish municipalities; Askø, Bandholm, Birket, Branderslev, Fejø, Femø, Gloslunde-Græshave, Herredskirke-Løjtofte, Hillested, Holeby, Horslunde-Nordlunde, Hundseby, Højreby, Købelev, Maribo købstads landdistrikt, Nebbelunde-Sædinge, Ringsebølle, Rudbjerg, Sandby, Stokkemarke, Tirsted-Skørringe-Vejleby, Tågerup-Torslunde, Utterslev, Vesterborg, Vindeby and Østofte. Guldborgsund municipality was identified as the provincial cities; Nykøbing Falster, Nysted, Sakskøbing and Stubbekøbing, and the parish municipalities; Døllefjelde-Musse, Falkerslev, Gundslev, Herritslev, Horbelev, Idestrup, Karleby-Horreby-Nr. Ørslev, Kettinge-Bregninge, Kippinge-Brarup-Stadager, Maglebrænde, Majbølle, Nysted sogn, Nr. Alslev-Nr. Kirkeby, Nr. Vedby, Radsted, Sakskøbing landsogn, Skelby-Gedesby, Slemminge-Fjelde, Systofte, Sdr. Kirkeby-Sdr. Alslev, Tingsted, Toreby, Torkildstrup-Lillebrænde, Tårs, V. Ulslev, Vigsnæs, Væggerløse, Våbensted-Engestofte, Vålse, Ønslev-Eskilstrup, Ø. Ulsted-Godsted and Åstrup. In 1970–2006, Lolland municipality was defined as the municipalities; Holeby, Højreby, Maribo, Nakskov, Ravnsborg, Rudbjerg and Rødby. Guldborgsund municipality was defined as the municipalities; Sakskøbing, Stubbekøbing, Sydfalster, Nykøbing-Falster, Nysted and Nørre Alslev. From 2007 onwards, the area has been divided into the two municipalities Lolland and Guldborgsund

Lolland-Falster is administratively divided into two municipalities: Lolland (western part of Lolland-Falster) and Guldborgsund (eastern part) (Fig. 1).

Annual population numbers 1971–2018 by sex and 5-year age groups for Denmark and for Lolland and Guldborgsund municipalities were extracted from the Statistics Denmark database “StatBank Denmark”. For 1970, numbers by sex and age were extracted manually from published data. For 1968 and 1969, numbers by sex and age for all of Denmark, and total population number for the two municipalities were extracted manually from published data (Table 1).

**Table 1:** Data sources for population and number of deaths at municipality and national level

Year	Population Number	Number of Deaths
1968	Befolkningens Bevægelser 1968 [Population Movements 1968]. Copenhagen; 1970	
1969	Befolkningens Bevægelser 1969 [Population Movements 1969]. Copenhagen; 1971	
1970	Befolkningen i de enkelte kommuner pr. 1. maj 1970 - fordelt efter køn, alder og ægteskabelig stilling [The population of the individual municipalities per May 1, 1970 - by sex, age and marital status]. Statistisk Tabelværk 1970: V. Copenhagen; 1970	Befolkningens Bevægelser 1970 [Population Movements 1970]. Copenhagen; 1972
1971	Statistics Denmark. Population in Denmark [Internet]. StatBank Denmark. Available from: http://www.statistikbanken.dk	Befolkningens Bevægelser 1971 [Population Movements 1971]. Copenhagen; 1973
1972		Befolkningens Bevægelser 1972 [Population Movements 1972]. Copenhagen; 1974
1973		Befolkningens Bevægelser 1973 [Population Movements 1973]. Copenhagen; 1975
1974–2018		Statistics Denmark. Deaths [Internet]. StatBank Denmark. Available from: http://www.statistikbanken.dk

Annual numbers of deaths by sex and 5-year age groups for 1974–2017 for Denmark and the two municipalities were extracted from the StatBank Denmark. Annual number of deaths by sex and age for 1968–1973 for Denmark were extracted manually from published data, while for the municipalities annual numbers of deaths were available by sex only for 1970–1973, and as a total number only for 1968–1969.

Deaths by main cause of death for 1973–2017 were tabulated based on individual data retrieved from the Danish Central Population Register and the Danish Cause of Death Register. Nationally, cause of death was missing for 29,179 deaths; 230 from Lolland and 271 from Guldborgsund.

### Statistical analysis

Mortality rates were calculated by sex, 5-year age groups, and 5-year calendar periods 1968–1972, 1973–1977, … 2008–2012 and 2013–2017 for all of Denmark, and for 1974–1977, 1978–1982, … 2008–2012 and 2013–2017 for Lolland and Guldborgsund municipalities, as the number of deaths divided by the number of persons-years from which the number of deaths were generated. For 1968–1969, break-down of person-years by sex and age in the municipalities was estimated from the national distribution, and the same was true for break-down of death by sex for the municipalities in 1968–1969. The oldest age interval was reported as ≥95 years in 1978–2017, ≥90 years in 1973–1977, and ≥80 years in 1968–1972.

Life expectancy was calculated by sex and 5-year calendar periods 1974–1977, 1978–1982, … 2008–2012 and 2013–2017 (first calendar period was only 4 years) for Lolland and Guldborgsund municipalities separately, and for all of Denmark. The life table method, as described by Fergany [15], was used with mortality rates for 5-year age groups (including < 1 years, 1–4 years and ≥95 years) applied in the life table.

Standardised mortality ratio (SMR) was calculated by sex and 5-year calendar period 1968–1972, 1973–1977, … 2008–2012 and 2013–2017 for Lolland and Guldborgsund municipality separately, using Denmark as the reference population. To calculate the SMR, the observed number of deaths by sex, calendar period and municipality was divided by the corresponding number of expected deaths calculated from summarizing across the 5-year age groups, the person-years multiplied by the Danish death rate.

$$ {SMR}_{spm}=\frac{{Death s}_{spm}}{\sum \limits_a\left( Person-{years}_{aspm}\ast {Death\ rate}_{aspDK}\right)} $$

where a=5-year age group; s=sex; *p*=5-year calendar period; m=municipality; and DK=Denmark. 95% confidence intervals (CI) were calculated under the assumption of a Poisson distribution of the observed number of deaths [16].

Furthermore, crude mortality ratios for 10-year age groups from 20 to 29 to 90+ years were calculated by sex and 5-year calendar period for Lolland and Guldborgsund municipalities separately, using Denmark as the reference population.

In order to consider a possible effect of birth cohorts on mortality, age-period-cohort analysis was conducted by organizing the dataset into synthetic 5-year birth cohorts based on the 5-year age groups and 5-year calendar periods. Poisson regression was used to estimate the rate ratio (RR) of death. RRs for 5-year calendar periods using 1974–1979 as reference were adjusted for birth cohort and age; RRs for 5-year birth cohorts using 1942–1946 as reference were adjusted for calendar period and age, and RRs for 5-year age groups using 0–4 years as reference were adjusted for calendar period and birth cohorts. Age-period-cohort analysis were made separately for men and women and for the total Danish population, Lolland and Guldborgsund municipalities, respectively.

Finally, SMRs were calculated for four mutually exclusive main causes of death; cancer, cardiovascular diseases, other natural causes, and external causes.

As the age-specific numbers of deaths were available on the municipality level only from 1974 onwards, the life expectancy, crude mortality ratios for 10-year age groups, and age-period-cohort analysis were calculated from 1974 onwards. SMR’s could be calculated from 1968 onwards.

## Results

**Fig. 2:**
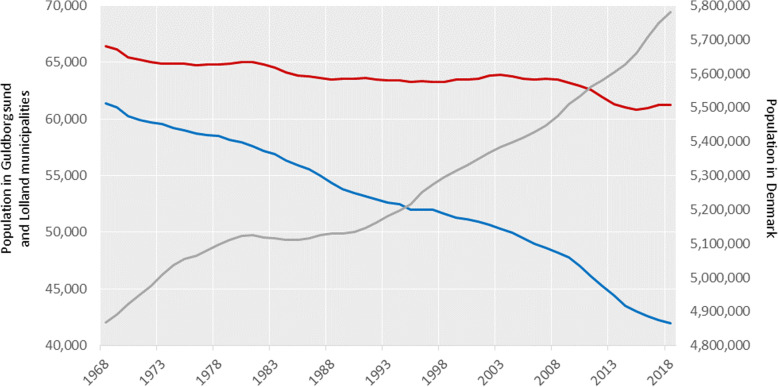
Population in Lolland and Guldborgsund municipalities and Denmark, 1968–2018

Between 1968 and 2018, the population of Lolland municipality decreased by 31% for men and 32% for women, and in Guldborgsund municipality by 8 and 7%, respectively (Fig. 2 and Additional file 1, Table S1). This decrease occurred in the age groups below 45 years, while the number of people above age 65 years increased (Additional file 1, Fig. S1). In Denmark, the population increased by 19% for men and 18% for women between 1968 and 2018, so the population of Lolland-Falster represented 2.6% of the Danish population in 1968, and 1.8% in 2018.

**Fig. 3:**
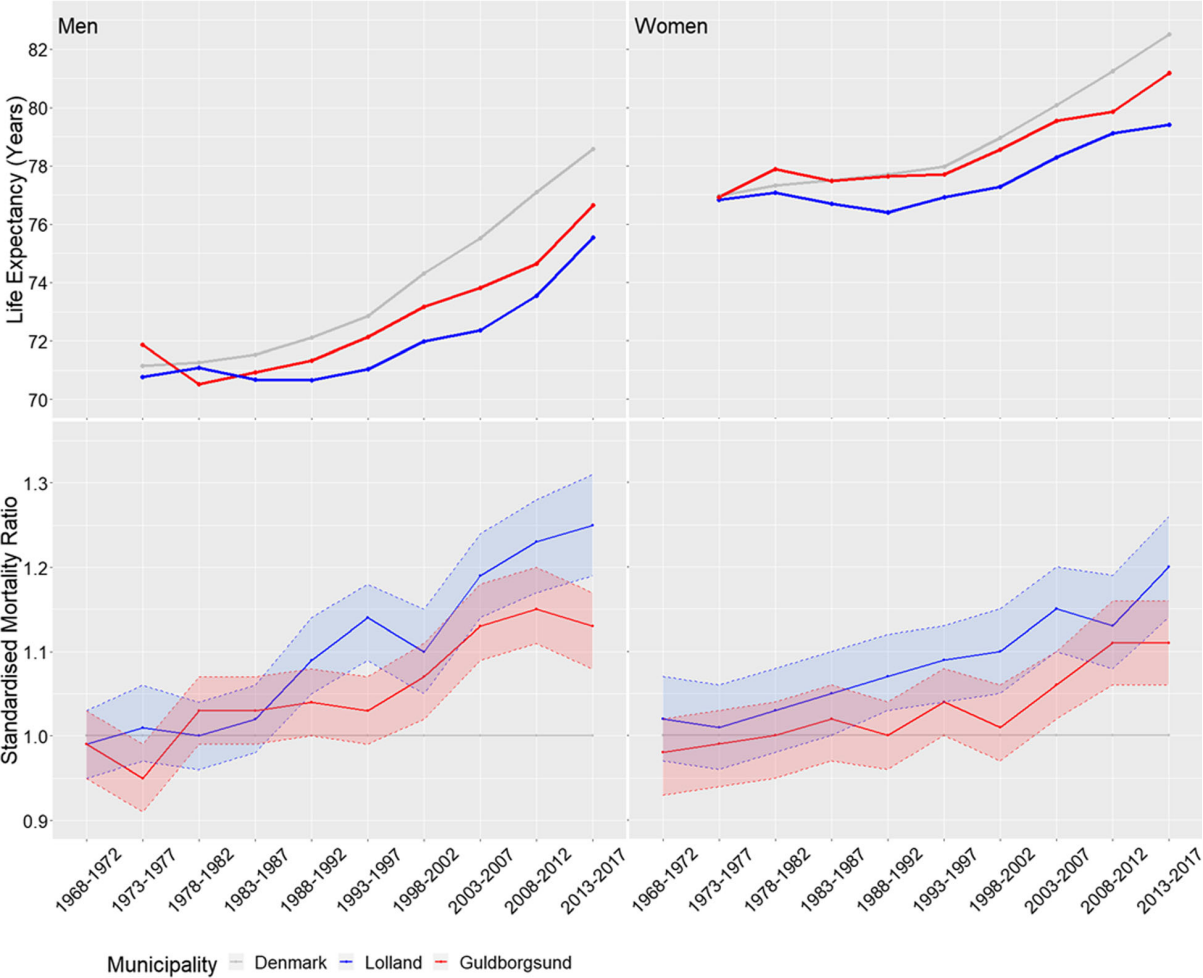
Above: Life expectancy for Lolland and Guldborgsund municipalities and Denmark by sex and 5-year calendar periods 1974–2017 (first calendar period is only 4 years, 1974–1977). Below: Standardised mortality ratios for Lolland and Guldborgsund municipalities by sex and 5-year calendar periods 1968–2017 (standard population is Denmark)

In 1974–1977, the life expectancy for men in Denmark was 71.14 years, while it was 70.76 and 71.87 years for men in Lolland and Guldborgsund municipalities, respectively. For women, the life expectancy in 1974–1977 was 76.97 years in Denmark, 76.82 years in Lolland municipality and 76.93 years in Guldborgsund municipality. This means that in 1974–1977, the life expectancy in Lolland and Guldborgsund municipalities was fairly similar to the national average (Fig. 3 and Additional file 1, Table S2).

In 2013–2017, the life expectancy for men had increased to 78.58 years in Denmark, and to 75.55 and 76.64 years in Lolland and Guldborgsund municipalities, respectively. The numbers for women were 82.51, 79.41 and 81.18 years, respectively. This means that from 1974 to 2017, the life expectancy had increased in Lolland and Guldborgsund municipalities, but less so than on the national basis.

During the period 1968–1987, both men and women in Lolland municipality had an overall mortality similar to that of the total Danish population with SMRs in the range of 0.99 to 1.05 (Fig. 3 and Additional file 1, Table S2). The same pattern was seen for men in Guldborgsund municipality in 1968–1997, and for women in 1968–2002. From 1988 onwards the mortality in Lolland municipality differed increasingly from that of Denmark; with the SMR for men being 1.25 (95% CI 1.19–1.31) and for women being 1.20 (1.14–1.26) in 2013–2017. A similar, but less marked and later, trend was seen for Guldborgsund municipality, where the SMR for men increased from 1.07 (95% CI 1.02–1.11) to 1.15 (95% CI 1.11–1.20) in 1998–2017, and for women from 1.06 (95% CI 1.02–1.10) to 1.11 (95% CI 1.06–1.16) in 2003–2017.

**Fig. 4:**
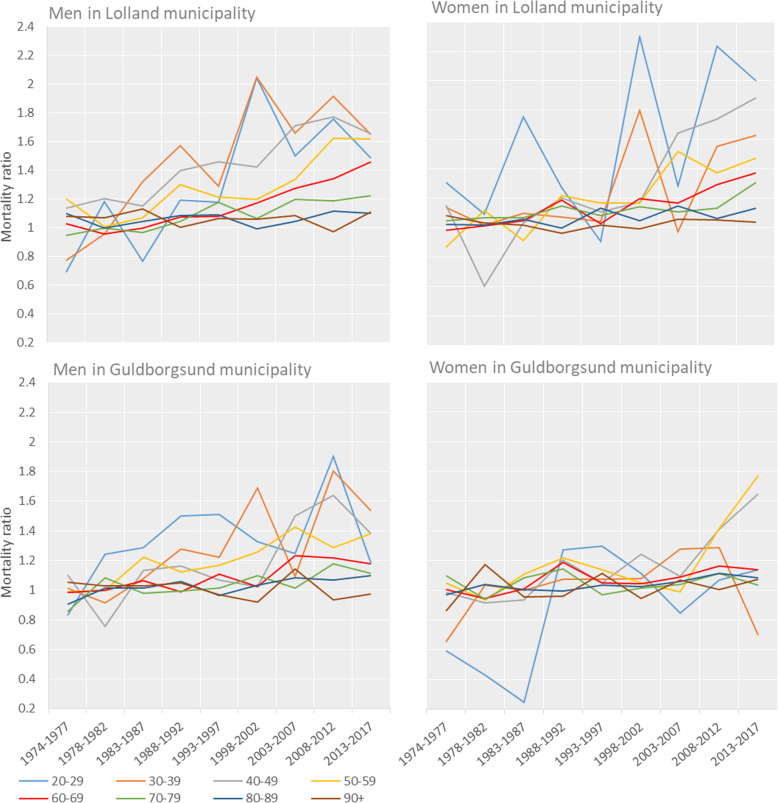
Crude mortality ratio for 10-year age groups and 5-year calendar periods 1974–2017 (first calendar period is only 4 years, 1974–1977). For Lolland and Guldborgsund municipalities by sex (reference population = total population of Denmark)

The mortality ratios for 10-year age groups showed that the relatively increased mortality in Lolland and Guldborgsund municipalities was mainly in the age groups between 20 and 69 years (Fig. 4). Men and women aged ≥70 years in Lolland and Guldborgsund municipalities had a mortality similar to that of the total Danish population.

The age-period-cohort analysis showed an overall decrease in RR across birth cohort and calendar period, and an increase with age (Additional file 1, Fig. S2 and S3). For calendar period, the decrease in mortality accelerated around 1993–1997 in all of Denmark, in Lolland, and in women, but not in men, in Guldborgsund. For men born in the 1930s and the 1940s, mortality stagnated in all of Denmark, and a similar pattern was seen in Lolland and Guldborgsund although encompassing a broader range of birth cohorts born from the 1920s to the first half of the 1950s. A somewhat similar, but less clear, cohort pattern was seen for women. It should be noted that all municipality estimates had relatively broad confidence intervals.

Deaths from cancer, cardiovascular disease, other natural, and external causes of death, all contributed to the increasing gap in mortality between Lolland-Falster and Denmark (Additional file 1, Fig. S4). Cancer mortality contributed to the gap only from 2008 to 2012. In Lolland, cardiovascular mortality contributed to the gap already from 1988 to 1992, reaching an SMR of 1.33 (95% CI 1.21–1.45) for men and 1.26 (95% CI 1.14–1.39) for women in 2013–2017. In Guldborgsund, cardiovascular mortality contributed to the gap from 1998 to 2002 for men, and from 2008 to 2012 for women, reaching an SMR of 1.14 (95% CI 1.05–1.24) for men and 1.19 (95% CI 1.09–1.29) for women in 2013–2017. The development was less regular for other natural causes of death with a peak for men in 2003–2007, SMR 1.28 (95% CI 1.19–1.38) for Lolland, and 1.21 (95% CI 1.13–1.29) for Guldborgsund. For these other natural causes of death, the SMR for women in Lolland increased from 1988 to 1992 reaching SMR 1.19 (95% CI 1.11–1.28) in 2013–2017, while for women in Guldborgsund, the SMR fluctuated and was at unity in 2013–2017. For external causes of death, the SMRs fluctuated over time with a tendency of increased SMRs for men in both municipalities, and for women in Lolland.

## Discussion

### Main findings

In the 1970s, the life expectancy in Lolland and Guldborgsund municipalities was similar to the Danish average. However, fifty years later in the 2010s, the life expectancy for both men and women in Lolland municipality lagged 3 years behind the Danish average, and in Guldborgsund municipality 2 years for men and 1 for women. These changes started in the 1980s in Lolland municipality and in the 1990s in Guldborgsund municipality and were due to mortality rates above the national average in young and middle-aged people. The presently high mortality of people in Lolland-Falster compared with that of the total population of Denmark was thus a new phenomenon, and not an inherent part of life in the area. Age-period-cohort analysis for all of Denmark pointed to a stagnation in mortality for persons born from around 1930 to 1950. For Lolland and Guldborgsund municipalities this stagnation seemed to encompass a broader range of cohorts, but the municipality data came with some statistical uncertaincy. Mortality from cardiovascular disease followed the pattern of the all-cause mortality, while smaller and less regular contributions were seen for mortality from cancer, other natural, and external causes of death.

### Previous studies

In the European Union, a comparison across 129 regions showed that the absolute gap between the longest and the shortest life expectancy remained constant from 1991 to 2008, though within Eastern Europe an increasing gap was observed among men [17]. However, several other countries have discovered increasing geographical difference in mortality. In England, an increased disparity in mortality was observed between the north and the south from 1965 to 2015 [18]. Increasing inequality in life expectancy was found between districts in New Zealand from 1980 to 2001 [19]. In contrast, a study of the 19 Norwegian counties found limited changes in mortality differences between 1980 and 2014; for men a decreasing gap in life expectancy (5.2 to 4.6 years), and for women an increasing gap (3.4 to 3.9 years) [20]. In Russia, men in Moscow in 2003 lived 7.1 years longer than men in Russia as a whole; while by 2014 this gap had increased to 8.3 years. The same trend was seen for men in St. Petersburg, where men in 2003 lived 3.0 years longer than men in Russia as a whole; this difference had increased to 5.3 years by 2014 [21].

In older studies from Denmark, Hoem found the mortality in Storstrøm county (including Lolland-Falster) in 1970–1979 to be fairly similar to the national average [22]. Throughout the period from 1970 to 2019, the capital Copenhagen had a life expectancy below that of other geographical areas of Denmark, however, since the 1980s, the life expectancy of Copenhagen approached the national average (Additional file 1, Fig. S5). The opposite trend was seen for West and Southern Zealand, which includes Lolland-Falster, were the life expectancy increasingly fell behind that of the other geographical areas of Denmark. The area including Lolland-Falster, was the only one with a trend falling behind that of the national average.

### Interpretation

During the past 50 years, mortality has decreased all over Denmark. However, the decrease has been slower in Lolland-Falster than in the rest of Denmark, which has given rise to the development of a mortality gap. As the increased mortality in Lolland-Falster compared with that in the rest of Denmark is a new phenomenon, it is necessary to consider it in perspective of changes over time in the living conditions and economic development. The Organisation for Economic Co-operation and Development (OECD) reported an increase in income inequality between 1985 and 2013 for several OECD-countries, including Denmark [23]. OECD attributed this to several labour market changes, such as the decline in need for manual labour, international competition, outsourcing of production, and decreased income redistribution via taxes [23].

The Lolland-Falster labour market was historically dominated by farming and industrial production, especially industries related to agriculture. Farming and processing of sugar beets began in Lolland-Falster in the 1870s [24]. Still today, the only two sugar factories in Denmark are located in Lolland-Falster [25]. After World War II the heavy increasing agricultural mechanization and technological progress made a largescale workforce redundant. An example is the availability of monogerm sugar beet seeds from 1969 [26] eliminated the labour intensive singling of plants in the sugar beet fields, and better capacity for transportation of sugar beets reduced the need for local processing facilities [27]. Therefore, the need for manual labour in farming decreased over time.

During the 1970s, the agricultural products processing industries and derivated industries such as machine factories were centralized and closed [28]. Simultaneously, a global shipyard crisis in the 1970s and 1980s resulted in the closure of Nakskov Shipyard in 1986 which in the 1970s was the largest workplace in Lolland-Falster with around 2000 employees [29]. In 1988, a cereal production site, the second largest workplace in Nakskov, closed [30]. The closure of several large and medium-sized industries from 1970 and especially the closure of the shipyard made many highly skilled workers redundant, and as they had to seek work outside Lolland-Falster, this started the decrease in number of inhabitants of working ages.

An administrative reform in Denmark in 2007 reduced the number of municipalities from 271 to 98, making Denmark one of the countries in Europe with the highest average number of inhabitants per municipality [31]. In Lolland-Falster, 13 municipalities were merged into two. Since the reform, Lolland-Falster among other marginal areas of Denmark, has experienced a decrease in the number of jobs in the public sector, especially jobs requiring a higher education, while the opposite has been seen in the large cities of Copenhagen and Aarhus [32].

The Danish Economic Council of Labour Market found that from 1990 to 2015, the net percentage of employed people aged 25–64 years moving away from Lolland municipality was 19%, while the net percentage of unemployed people moving to Lolland municipality was 4% [33]. In 2019, Statistics Denmark found that Lolland and Guldborgsund were among the municipalities with the lowest percentage of people aged 25–65 with completed vocational training/higher education, while the highest percentage was found in the municipalities around Copenhagen and Aarhus [34]. Elderly people were left behind as the working-age population decreased in Lolland-Falster (Additional file 1, Fig. S1), and as in other fringe municipalities of Denmark, house prices increased considerably less than in urban municipalities [35].

The decrease in number of work places in agriculture, manufacture and public administration affected also the social life, as much of the spare time and support activities were organised by agricultural associations [36] and union clubs [37]. These societal changes have been illustrated in art, e.g. in the film “The last song of Mifune” [38], in photographic documentation as “Home” [39], in autobiographies like “Zornig - anger is my middle name” [40], and in novels like “Rødby-Puttgarden” [41], and scientifically documented in narratives [42].

However, this gradual depletion of the social capital has not been well documented statistically. This might in part be due to the complexity of the Danish welfare state, where the unemployment rate reflects only partially the social situation, and where routine statistics will not take into account that a given person can receive different types of benefits. Register-based research is needed to address these challenges. Using individual register data and taking 20 types of social benefit into account, the Danish Economic Council of Labour Market documented that while Lolland-Falster in 2016–2018 experienced a net inflow of 26% in persons on social benefit, the capital of Copenhagen in the same period experienced a net outflow of 22% [43]. Survey data on self-reported health are also available only from more recent years. In the Danish National Health Profile from 2010, 2013, and 2017 [44], inhabitants from Lolland-Falster scored high on risk factors such as smoking, overweight, psychological stressors, and lack of exercise.

Mortality from cardiovascular diseases was until 2009 the major contributor to all-cause mortality in Denmark where after it was surpassed by cancer [45], and the trend for mortality from cardiovascular diseases is therefore expected to follow the trend for all-cause mortality. Furthermore, it has been described previously that in Denmark social differences in mortality were larger for cardiovascular diseases than for cancer [46]. High body mass index, high blood pressure, and smoking are well established risk factors for cardiovascular diseases, but it has been demonstrated that even after adjustment for these risk factors, cardiovascular diseases occur more frequently in people with low than in people with high education [47]. Lolland and Guldborgsund municipalities have throughout the study period been served by a local hospital including also a cardiology department. As in all of Denmark, admittance to hospital is free of charge for the patient. Although it cannot be excluded that regional differences in health care can have contributed to the regional differences in mortality from cardiovascular diseases, it is more likely that these differences are related to differences in disease occurrence attributable to body mass index, blood pressure, smoking and social status.

It has been demonstrated previously that the inequalities in health across educational groups are not smaller in the Nordic countries than in other European countries. Recently the mortality difference across educational groups even tended to widen [48]. In Denmark, the quartile of men with the highest education increased their life expectancy by 5.3 years from 1987 to 2011, while the increase was only 3.3 years for men in the quartile with the lowest education; and the corresponding changes for women were 3.7 years and 2.4 years, respectively [49].

The case of Lolland-Falster illustrates what Mackenbach called the Nordic paradox, where welfare policies including tax-paid unemployment benefits, and free education and health care have not been able to counterbalance the inequalities generated by macroeconomic changes [50]. In this perspective, the development in Denmark follows to some extent the pattern seen in the US, where increasing inequality in living conditions was followed by increasing inequality in mortality. In the Danish setting the mortality trends of people of working-age in the marginalized area Lolland-Falster are, however, still unfavourable in relative terms only, as the life expectancy has increased also in Lolland-Falster.

### Study limitations

Proportionality between the compared mortality rates across age groups is a condition for calculation of age-standardised mortality indicators. This condition was fulfilled up until 1988, thereafter in particular the mortality for persons aged 20–69 years started to differ from this pattern. However, the correspondence between changes over time in life expectancies and SMRs supports the robustness of our results.

We interpreted the trend in mortality in Lolland-Falster in the framework of major societal changes in the area, but an in-depth analysis of the economic and structural changes in Lolland-Falster over the past 50 years was beyond the scope of the present study.

## Conclusion

The study showed that the presently low life expectancy and high mortality in Lolland-Falster compared with the rest of Denmark was a relatively new phenomenon. The mortality in Lolland municipality was at the level of the national average up until 1988, and in Guldborgsund municipality up until 1998 for men and 2003 for women. The increasing gap in mortality between Lolland-Falster and Denmark in general coincided with changes in the economic structure in Lolland-Falster where the mechanisation of agricultural production resulted in a decreasing need for manual labour; the recession during the 1970s and 1980s with closure of companies, especially the shipyard, made many highly skilled workers redundant, and they had to seek jobs outside Lolland-Falster. Finally, an administrative reform in 2007 led to centralization of public institutions with the disappearance from the area of many jobs requiring higher education. Our study is the first one to report on a significant increasing geographical segregation in all-cause mortality in a Nordic welfare state.

## Supplementary Information

**Additional file 1: Table S1**. Mean population and mean number of deaths per year by sex for 5-year periods in Lolland municipality, Guldborgsund municipality and Denmark. **Table S2**. Life expectancy (years) and standardised mortality ratios [95% confidence interval] for Lolland and Guldborgsund municipalities by sex and 5-year calendar period. The standard population is Denmark. **Figure S1**. Population number for Lolland and Guldborgsund municipalities and for the total Danish population, 1968–2018. Age groups 0–24, 25–44, 45–64. **Figure S2**. Age-period-cohort analysis as rate ratios for men in Denmark, Lolland and Guldborgsund municipalities. Estimates for year adjusted for age and birth cohort using 1974–77 as reference. Estimates for birth cohort adjusted for age and year using 1942–46 as reference. Estimates for age adjusted for year and birth cohort using 0–4 years as reference. **Figure S3**. Age-period-cohort analysis as rate ratios for women in Denmark, Lolland and Guldborgsund municipalities. Estimates for year adjusted for age and birth cohort using 1974–77 as reference. Estimates for birth cohort adjusted for age and year using 1942–46 as reference. Estimates for age adjusted for year and birth cohort using 0–4 years as reference. **Figure S4**. Standardised mortality ratio by cause of death for Lolland and Guldborgsund municipalities by sex (reference population = total population of Denmark). **Figure S5**. Life expectancy of geographical areas in Denmark for 10-year periods, 1970–2019.

## Data Availability

All data are publicly available online open access from Statistics Denmark reports and StatBank Denmark (Table 1). Data can be accessed from: • Statistics Denmark. Befolkningens Bevægelser 1968 [Population Movements 1968]. Copenhagen; 1970. Available from: https://www.dst.dk/Site/Dst/Udgivelser/GetPubFile.aspx?id=19766&sid=folk1968 • Statistics Denmark. Befolkningens Bevægelser 1969 [Population Movements 1969]. Copenhagen; 1971. Available from: https://www.dst.dk/Site/Dst/Udgivelser/GetPubFile.aspx?id=19765&sid=folk1969 • Statistics Denmark. Befolkningens Bevægelser 1970 [Population Movements 1970]. Copenhagen; 1972. Available from: https://www.dst.dk/Site/Dst/Udgivelser/GetPubFile.aspx?id=19764&sid=folk1970 • Statistics Denmark. Befolkningen i de enkelte kommuner pr. 1. maj 1970 - fordelt efter køn, alder og ægteskabelig stilling [The population of the individual municipalities per may 1, 1970 - by sex, age and marital status]. Stat. Tabelværk 1970 V. Copenhagen; 1970. Available from: https://www.dst.dk/Site/Dst/Udgivelser/GetPubFile.aspx?id=20964&sid=befkom1970 • Statistics Denmark. Befolkningens Bevægelser 1971 [Population Movements 1971]. Copenhagen; 1973. Available from: https://www.dst.dk/Site/Dst/Udgivelser/GetPubFile.aspx?id=19763&sid=folk1971 • Statistics Denmark. Befolkningens Bevægelser 1972 [Population Movements 1972]. Copenhagen; 1974. Available from: https://www.dst.dk/Site/Dst/Udgivelser/GetPubFile.aspx?id=19762&sid=folk1972 • Statistics Denmark. Befolkningens Bevægelser 1973 [Population Movements 1973]. Copenhagen; 1975. Available from: https://www.dst.dk/Site/Dst/Udgivelser/GetPubFile.aspx?id=19761&sid=folk1973 • Statistics Denmark. Population in Denmark [Internet]. StatBank Denmark. Available from: http://www.statistikbanken.dk/statbank5a/default.asp?w=1920 • Statistics Denmark. Deaths [Internet]. StatBank Denmark. Available from: http://www.statistikbanken.dk/statbank5a/default.asp?w=1920

## Abbreviations

CI: Confidence interval
OECD: The Organisation for Economic Co-operation and Development
RR: Rate Ratio
SMR: Standardised mortality ratio

## References

[1] Oeppen J, Vaupel JW (2002). Broken limits to life expectancy. Science..

[2] The World Bank. Life expectancy at birth, total (years) - European Union [Internet]. Data. 2020 [cited 2020 Dec 23]. Available from: https://data.worldbank.org/indicator/SP.DYN.LE00.IN?end=2017&locations=EU&start=1960.

[3] Woolf SH, Schoomaker H (2019). Life expectancy and mortality rates in the United States, 1959-2017. JAMA..

[4] James W, Cossman J, Wolf JK (2018). Persistence of death in the United States: the remarkably different mortality patterns between America’s heartland and Dixieland. Demogr Res.

[5] Miyagi S, Iwama N, Kawabata T, Hasegawa K. Longevity and Diet in Okinawa, Japan: The Past, Present and Future. Asia Pac J Public Heal. 2003;15(Supp.):3–9. 10.1177/101053950301500S03 18924533

[6] Juel K (2004). Dødeligheden i Danmark gennem 100 år [Mortality in Denmark during 100 years].

[7] Christensen K, Davidsen M, Juel K, Mortensen L, Rau R, Vaupel JW, Crimmins EM, Preston SH, Cohen B (2010). The divergent life-expectancy trends in Denmark and Sweden—and some potential explanations. International differences in mortality at older ages.

[8] Wensink M, Alvarez JA, Rizzi S, Janssen F, Lindahl-Jacobsen R (2020). Progression of the smoking epidemic in high-income regions and its effects on male-female survival differences: a cohort-by-age analysis of 17 countries. BMC Public Health.

[9] Eurostat. Life expectancy at birth by sex [Internet]. EC data browser. 2020 [cited 2020 Dec 23]. Available from: https://ec.europa.eu/eurostat/databrowser/view/sdg_03_10/default/bar?lang=en.

[10] Statistics Denmark. Life expectancy for new born babies by region and sex [Internet]. StatBank Denmark. 2020 [cited 2020 Dec 23]. Available from:https://www.statbank.dk.

[11] Statistics Denmark. Denmark in figures 2019. Copenhagen; 2019.

[12] Helliwell, J. Helliwell, J., Layard, R., & Sachs J (2018). WHR 2018. NY, Layard R, Sachs J. World Happiness Report 2018. Sustainable development solutions network, N Y; 2018.

[13] World Health Organization (1946). Constitution of the World Health Organization.

[14] Statistics Denmark. Life expectancy for new born babies by municipality [Internet]. StatBank Denmark. 2020 [cited 2020 Dec 23]. Available from:https://www.statbank.dk.

[15] Fergany N (1971). On the human survivorship function and life table construction. Demography..

[16] SAS Institute. Indirect Standardization and Standardized Morbidity/Mortality Ratio [Internet]. SAS/STAT(R) 13.2 User’s Guide. 2019 [cited 2019 Sep 24]. Available from: http://support.sas.com.

[17] Richardson EA, Pearce J, Mitchell R, Shortt NK, Tunstall H (2014). Have regional inequalities in life expectancy widened within the European Union between 1991 and 2008?. Eur J Pub Health.

[18] Buchan IE, Kontopantelis E, Sperrin M, Chandola T, Doran T (2017). North-south disparities in English mortality 1965-2015: longitudinal population study. J Epidemiol Community Health.

[19] Pearce J, Dorling D (2006). Increasing geographical inequalities in health in New Zealand, 1980-2001. Int J Epidemiol.

[20] Skaftun EK, Verguet S, Johansson KA, Norheim OF (2018). Geographic health inequalities in Norway : a Gini analysis of cross-county differences in mortality from 1980 to 2014. Int J Equity Health.

[21] Timonin S, Danilova I, Andreev E, Shkolnikov VM (2017). Recent mortality trend reversal in Russia: are regions following the same tempo?. Eur J Popul.

[22] Hoem B (1983). Regionale dødelighedsforskelle i Danmark 1971–79 [Regional mortality differences in Denmark 1971–1979].

[23] Keeley B (2015). What’s happening to income inequality?. Income inequality: the gap between rich and poor.

[24] Bjørn C. Sukkerroer og Sukkerroedyrkning [Sugar Beet and Beet Cultivation]. In: Danske Sukkerroedyrkere i 100 år - 1902-2002 [Danish Sugar Beet Farmers for 100 years - 1902-2002]. Copenhagen: Danske Sukkerroedyrkere [Danish Sugar Beet Farmers]; 2002. p. 7–18.

[25] Nordic Sugar A/S. Vores fabrikker og kontorer [Our factories and offices] [Internet]. 2018 [cited 2018 Dec 19]. Available from: http://www.nordicsugar.dk.

[26] Maribo Seed. History [Internet]. 2017 [cited 2019 Sep 25]. Available from: http://www.mariboseed.com.

[27] Christensen H. Modernisering, diversifikation og fusionering 1945-ca. 2000 [Modernisation, diversification and merging 1945- about 2000]. In: Mygh L, Nordahl K, editors. Dansk Industri - sukkerfabrikkerne - roer i lange baner [Danish Industry - the sugar factories - beets in long lanes]. Nykøbing F.: Guldborgsund Museum; 2007. p. 117–28.

[28] Andersen ML, Buur LT. Perspektiver på industrisamfundets kulturarv i Storstrøms og Vestsjællands Amter [perspectives on the industrial society’s cultural heritage in the counties of Storstrøm and West Zealand] [internet]. Museumsrådene i Storstrøms og Vestsjællands Amter 2004. Available from: https://docplayer.dk/760824-Perspektiver-paa-industrisamfundets-kulturarv-i-storstroems-og-vestsjaellands-amter.html.

[29] Olesen TR. Lukningen af Nakskov skibsværft A/S i 1986 [The shutdown of Nakskov shipyard A/S in 1986]. In: Erhvervshistorisk årbog 2012, 2. Rigsarkivet; 2012. p. 1–31.

[30] Lunddrup D (2014). Nakskov - visioner og fakta [Nakskov - visions and facts].

[31] Lyck L (2014). Introduktion, problemstilling og analysetilgang [introduction, problem and analytical approach]. Udkantsdanmark og sammenhængskraft i Danmark - En helhedsanalyse [Marginal Denmark and cohesion in Denmark - An overall analysis].

[32] Damm EA, Salmon R (2017). I 2 ud af 3 kommuner er der færre offentligt ansatte i dag end i 2008 [in 2 out of 3 municipalities, there are fewer public employees today than in 2008].

[33] Juul JS, Blicher SP (2017). Folk i job fraflytter udkanten og folk uden job kommer til [people in jobs move away from margianl areas and people without jobs move in].

[34] Statistics Denmark. Store geografiske forskelle på uddannelsesniveau [Large geographical differences in educational level]. Nyt fra Danmarks Stat [News from Stat Denmark]. 2019;229.

[35] Hansen JZ, Østergaard A. Ejerboliger i det 21. århundrede [Owner-occupied homes in the 21st century]. Copenhagen; 2018. Available from: https://www.bvc.dk/faglige-udgivelser/ejerboliger-i-det-21-aarhundrede/.

[36] Frandsen JN. Landbokulturen fra storhed i 1950’erne til velfærdssamfund, kommunalreformer og perifer position [the rural culture from greatness in the 1950s to welfare societies, municipality reforms and peripheral position]. Landbohistorisk Tidsskr 2015;0–79.

[37] Christensen AG (2017). Min tid på værftet [my time at the ship yard].

[38] Kragh-Jacobsen S, Jensen AT (1999). Mifunes sidste sang [the last song of Mifune].

[39] Lose S. Home. Maribo: Storstrøms kunstmuseum; 2006. 119 p.

[40] Andersen LZ. Zornig - vrede er mit mellemnavn [Zornig - anger is my middlename]. 1st ed. Copenhagen: Gyldendal; 2011. 212 p.

[41] Helle H (2005). Rødby-Puttgarden.

[42] Ledstrup M. After precarity : a geography of dark news and digital hope on the island of Lolland. Cult Geogr. 2020:1–14.

[43] Juul JS, Blicher SP (2018). Offentligt forsørgede fraflytter de store byer [Persons on social support leave larger cities].

[44] Sundhedsstyrelsen & Statens Institut for Folkesundhed. Danskerne Sundhed [The health of the Danes] [Internet]. Tal fra Den Nationale Sundhedsprofil. 2017 [cited 2018 Oct 3]. Available from: http://danskernessundhed.dk/.

[45] Statistics Denmark. Deaths by sex, age, cause of death and time [Internet]. StatBank Denmark. 2020 [cited 2020 Dec 10]. Available from: https://www.statbank.dk.

[46] Toch-Marquardt M, Menvielle G, Eikemo TA, Kulhánová I, Kulik MC, Bopp M, et al. Occupational class inequalities in all-cause and cause-specific mortality among middle-aged men in 14 European populations during the early 2000s. PLoS One. 2014:9. 10.1371/journal.pone.0108072 PMC4182439 25268702

[47] Carter AR, Gill D, Davies NM, Taylor AE, Tillmann T, Vaucher J (2019). Understanding the consequences of education inequality on cardiovascular disease: Mendelian randomisation study. BMJ..

[48] Mackenbach JP, Rubio Valverde J, Bopp M, Brønnum-Hansen H, Costa G, Deboosere P (2019). Progress against inequalities in mortality: register-based study of 15 European countries between 1990 and 2015. Eur J Epidemiol.

[49] Brønnum-Hansen H, Baadsgaard M (2012). Widening social inequality in life expectancy in Denmark. A register-based study on social composition and mortality trends for the Danish population. BMC Public Health.

[50] Mackenbach JP (2017). Nordic paradox, southern miracle, eastern disaster: persistence of inequalities in mortality in Europe. Eur J Pub Health.

