# Supplementary material for: Emergence of a mortality disparity between a marginal rural area and the rest of Denmark, 1968-2017
Source: BMC Public Health. 2021 Jan 7;21:90. doi: 10.1186/s12889-020-10108-6 (PMC7791824; doi:10.1186/s12889-020-10108-6)
Supplement: Supplementary file 1 — Additional file 1: Table S1. Mean population and mean number of deaths per year by sex for 5-year periods in Lolland municipality, Guldborgsund municipality and Denmark. Table S2. Life expectancy (years) and standardised mortality ratios [95% confidence interval] for Lolland and Guldborgsund municipalities by sex and 5-year calendar period. The standard population is Denmark. Figure S1. Population number for Lolland and Guldborgsund municipalities and for the total Danish population, 1968–2018. Age groups 0–24, 25–44, 45–64. Figure S2. Age-period-cohort analysis as rate ratios for men in Denmark, Lolland and Guldborgsund municipalities. Estimates for year adjusted for age and birth cohort using 1974–77 as reference. Estimates for birth cohort adjusted for age and year using 1942–46 as reference. Estimates for age adjusted for year and birth cohort using 0–4 years as reference. Figure S3. Age-period-cohort analysis as rate ratios for women in Denmark, Lolland and Guldborgsund municipalities. Estimates for year adjusted for age and birth cohort using 1974–77 as reference. Estimates for birth cohort adjusted for age and year using 1942–46 as reference. Estimates for age adjusted for year and birth cohort using 0–4 years as reference. Figure S4. Standardised mortality ratio by cause of death for Lolland and Guldborgsund municipalities by sex (reference population = total population of Denmark). Figure S5. Life expectancy of geographical areas in Denmark for 10-year periods, 1970–2019. [file 12889_2020_10108_MOESM1_ESM.docx]

Nykøbing Falster Hospital 11 December 2020

**Supplementary material to: Holmager et al. Emergance of a mortality disparity between a marginal rural area and the rest of Denmark, 1968-2017.**

|  | **Lolland** | | | | **Guldborgsund** | | | | **Denmark** | | | |
| --- | --- | --- | --- | --- | --- | --- | --- | --- | --- | --- | --- | --- |
|  | Men | | Women | | Men | | Women | | Men | | Women | |
|  | Population | Deaths | Population | Deaths | Population | Deaths | Population | Deaths | Population | Deaths | Population | Deaths |
| 1968-1972 | 30438 | 416 | 30026 | 326 | 32935 | 457 | 32728 | 354 | 2439759 | 26217 | 2478468 | 22116 |
| 1973-1977 | 29580 | 435 | 29430 | 326 | 32408 | 455 | 32421 | 365 | 2501373 | 28121 | 2547292 | 23388 |
| 1978-1982 | 28861 | 435 | 29014 | 347 | 32220 | 510 | 32696 | 393 | 2525307 | 29687 | 2589434 | 25350 |
| 1983-1987 | 27774 | 439 | 28159 | 373 | 31501 | 503 | 32472 | 422 | 2520563 | 30286 | 2595590 | 27490 |
| 1988-1992 | 26525 | 455 | 27030 | 401 | 31305 | 502 | 32227 | 443 | 2533521 | 30511 | 2607087 | 29431 |
| 1993-1997 | 25896 | 454 | 26316 | 418 | 31269 | 496 | 32071 | 472 | 2577704 | 30598 | 2646121 | 30997 |
| 1998-2002 | 25523 | 402 | 25617 | 395 | 31381 | 473 | 32156 | 446 | 2634735 | 28560 | 2696469 | 29959 |
| 2003-2007 | 24775 | 404 | 24685 | 382 | 31510 | 473 | 32129 | 444 | 2678472 | 27338 | 2734947 | 28547 |
| 2008-2012 | 23496 | 380 | 23367 | 345 | 31173 | 446 | 31650 | 432 | 2742266 | 26480 | 2790359 | 27254 |
| 2013-2017 | 21713 | 356 | 21469 | 330 | 30450 | 425 | 30629 | 406 | 2816042 | 26275 | 2853078 | 26215 |

**Supplementary Table 1** Mean population and mean number of deaths per year by sex for 5-year periods in Lolland municipality, Guldborgsund municipality and Denmark.

|  | **Life Expectancy (Years)** | | | | | | **Standardised Mortality Ratio** | | | |
| --- | --- | --- | --- | --- | --- | --- | --- | --- | --- | --- |
|  | Lolland | | Guldborgsund | | Denmark | | Lolland | | Guldborgsund | |
|  | Men | Women | Men | Women | Men | Women | Men | Women | Men | Women |
| 1968-1972 | - | - | - | - | - | - | 0.99 [0.95-1.03] | 1.02 [0.97-1.07] | 0.99 [0.95-1.03] | 0.98 [0.93-1.02] |
| 1973-1977* | 70.76 | 76.82 | 71.87 | 76.93 | 71.14 | 76.97 | 1.01 [0.97-1.06] | 1.01 [0.96-1.06] | 0.95 [0.91-0.99] | 0.99 [0.94-1.03] |
| 1978-1982 | 71.09 | 77.07 | 70.52 | 77.88 | 71.26 | 77.31 | 1.00 [0.96-1.04] | 1.03 [0.98-1.08] | 1.03 [0.99-1.07] | 1.00 [0.95-1.04] |
| 1983-1987 | 70.67 | 76.68 | 70.92 | 77.48 | 71.54 | 77.50 | 1.02 [0.98-1.06] | 1.05 [1.00-1.10] | 1.03 [0.99-1.07] | 1.02 [0.97-1.06] |
| 1988-1992 | 70.67 | 76.41 | 71.32 | 77.65 | 72.12 | 77.71 | 1.09 [1.05-1.14] | 1.07 [1.03-1.12] | 1.04 [1.00-1.08] | 1.00 [0.96-1.04] |
| 1993-1997 | 71.04 | 76.92 | 72.15 | 77.71 | 72.85 | 77.96 | 1.14 [1.09-1.18] | 1.09 [1.04-1.13] | 1.03 [0.99-1.07] | 1.04 [1.00-1.08] |
| 1998-2002 | 71.97 | 77.28 | 73.16 | 78.57 | 74.32 | 78.97 | 1.10 [1.05-1.15] | 1.10 [1.05-1.15] | 1.07 [1.02-1.11] | 1.01 [0.97-1.06] |
| 2003-2007 | 72.35 | 78.29 | 73.83 | 79.54 | 75.53 | 80.09 | 1.19 [1.14-1.24] | 1.15 [1.10-1.20] | 1.13 [1.09-1.18] | 1.06 [1.02-1.10] |
| 2008-2012 | 73.55 | 79.12 | 74.66 | 79.85 | 77.11 | 81.25 | 1.23 [1.17-1.28] | 1.13 [1.08-1.19] | 1.15 [1.11-1.20] | 1.11 [1.06-1.16] |
| 2013-2017 | 75.55 | 79.41 | 76.64 | 81.18 | 78.58 | 82.51 | 1.25 [1.19-1.31] | 1.20 [1.14-1.26] | 1.13 [1.08-1.17] | 1.11 [1.06-1.16] |

**Supplementary Table 2** Life expectancy (years) and standardised mortality ratios [95% confidence interval] for Lolland and Guldborgsund municipalities by sex and 5-year calendar period. The standard population is Denmark.

*Life expectancy is only calculated for 1974-1977.

**Supplementary Figure 1** Population number for Lolland and Guldborgsund municipalities and for the total Danish population, 1968-2018. Age groups 0-24, 25-44, 45-64.

45-64 years

0-24 years

**Supplementary Figure 2** Age-period-cohort analysis as rate ratios for men in Denmark, Lolland and Guldborgsund municipalities. Estimates for year adjusted for age and birth cohort using 1974-77 as reference. Estimates for birth cohort adjusted for age and year using 1942-46 as reference. Estimates for age adjusted for year and birth cohort using 0-4 years as reference.


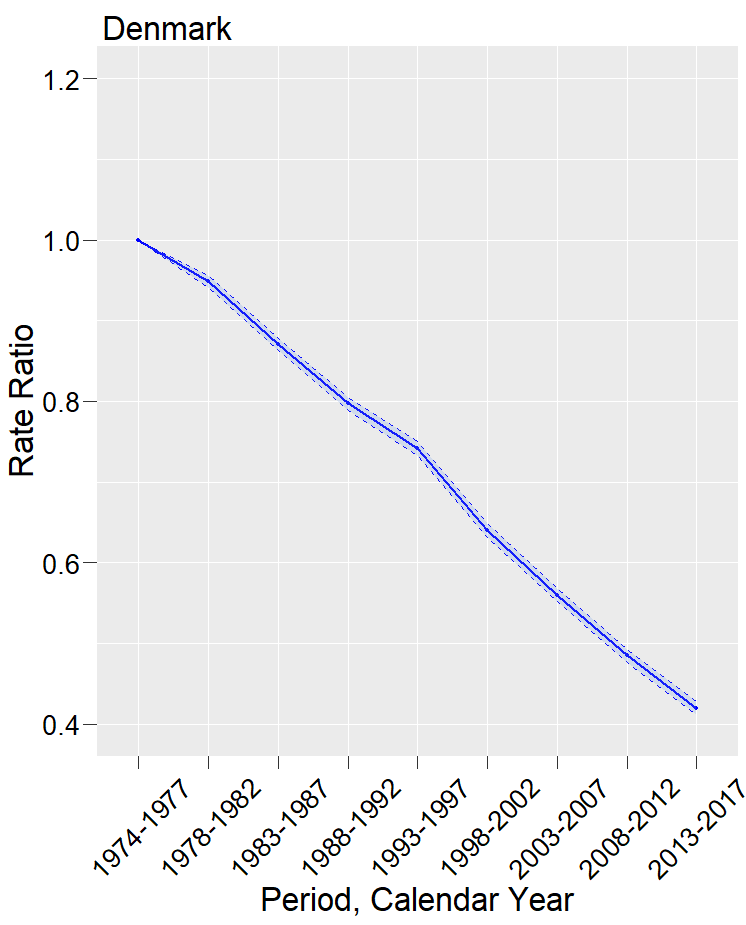

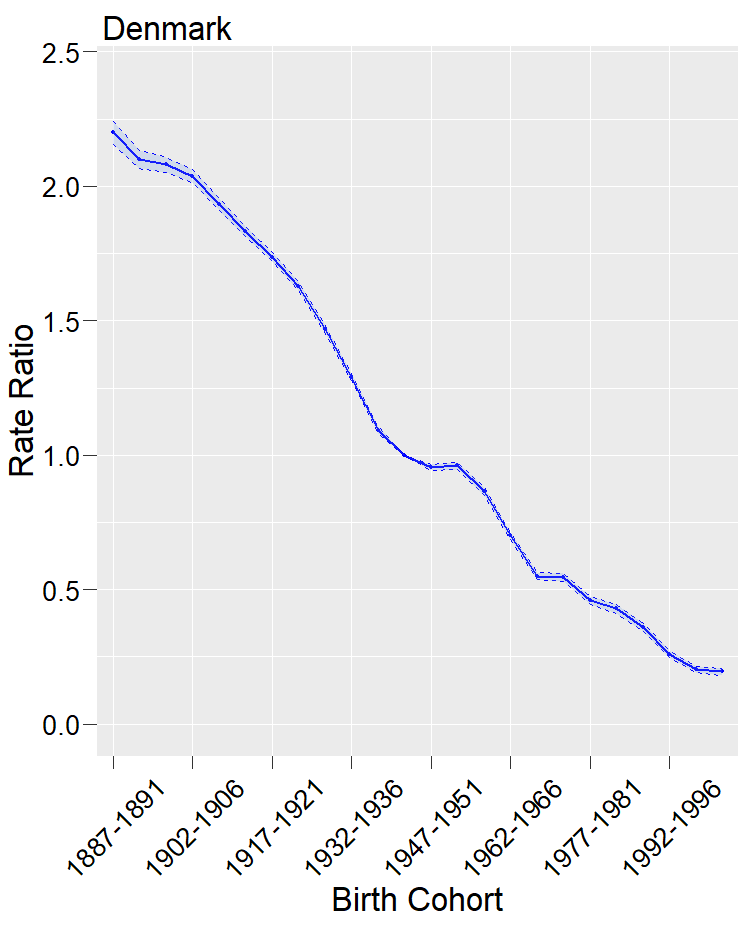

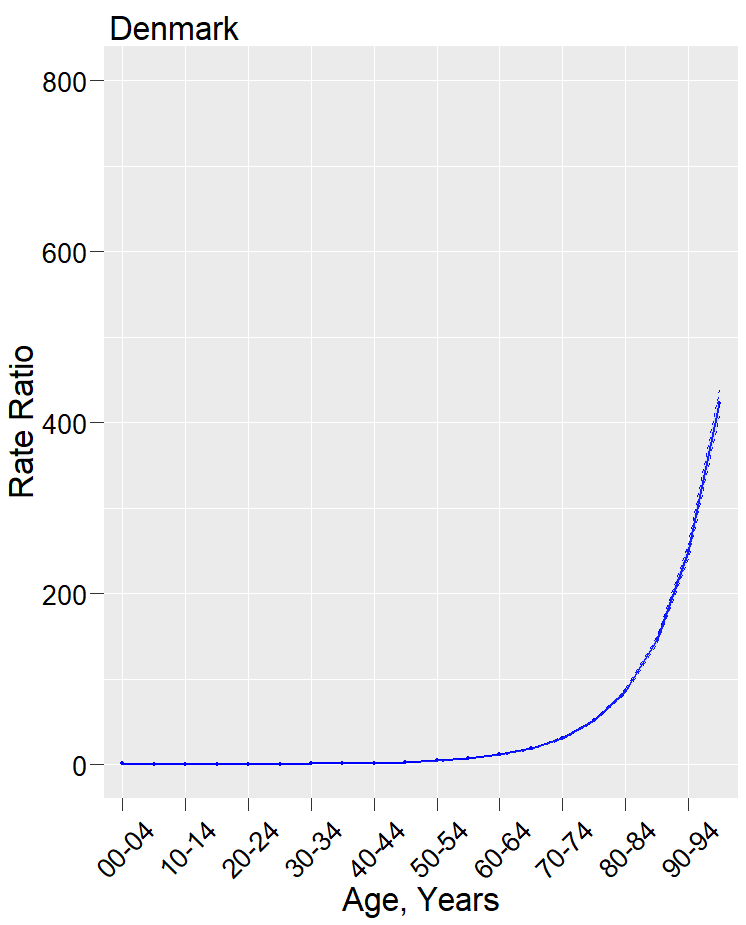

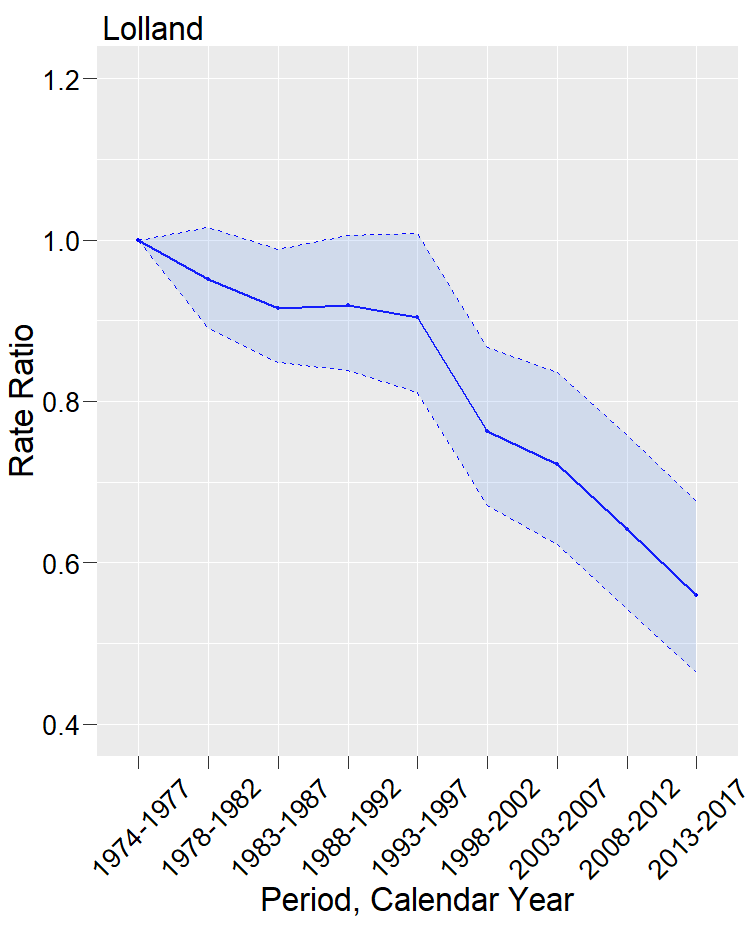

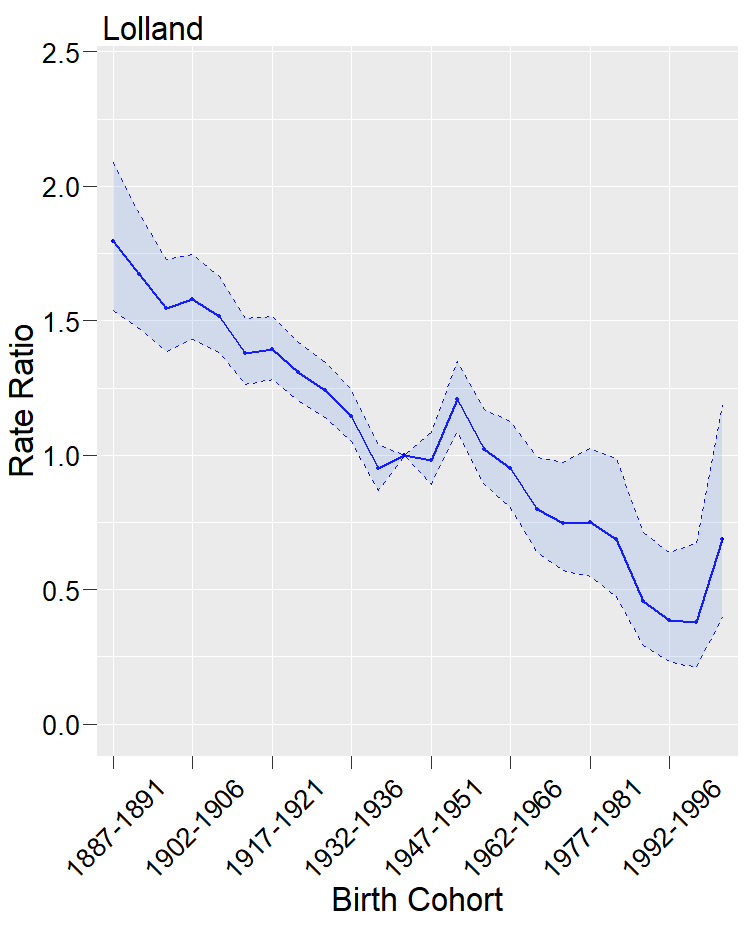

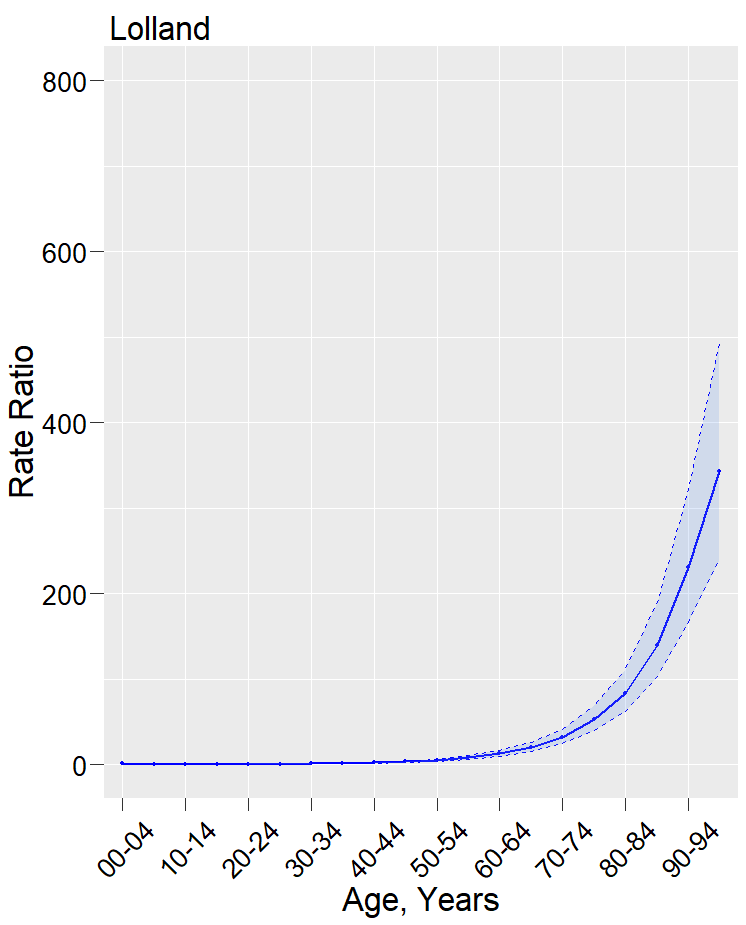


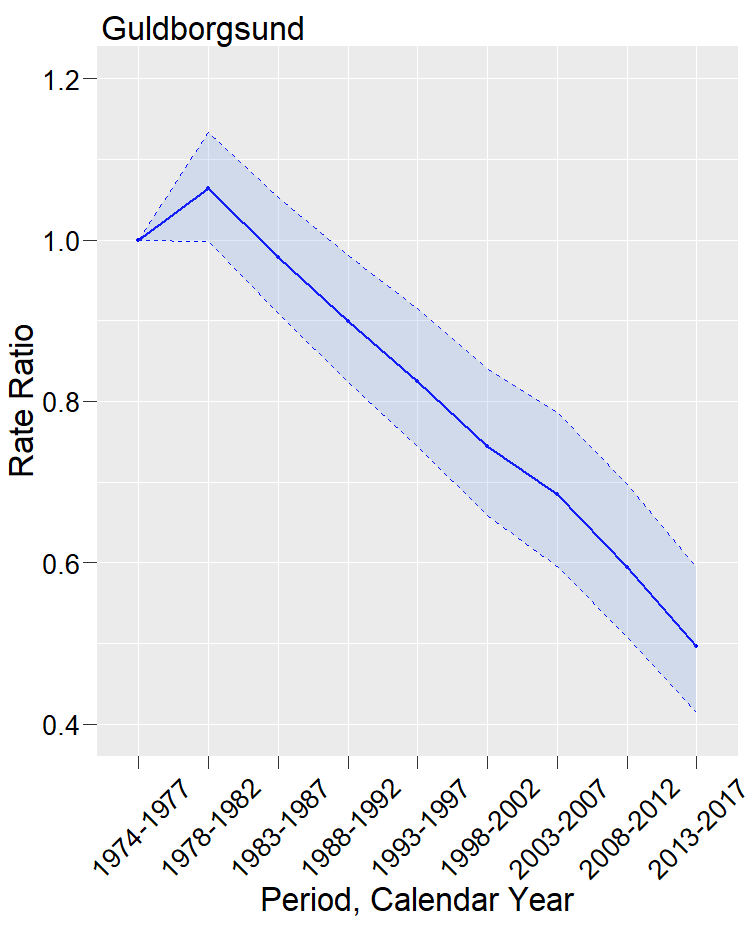

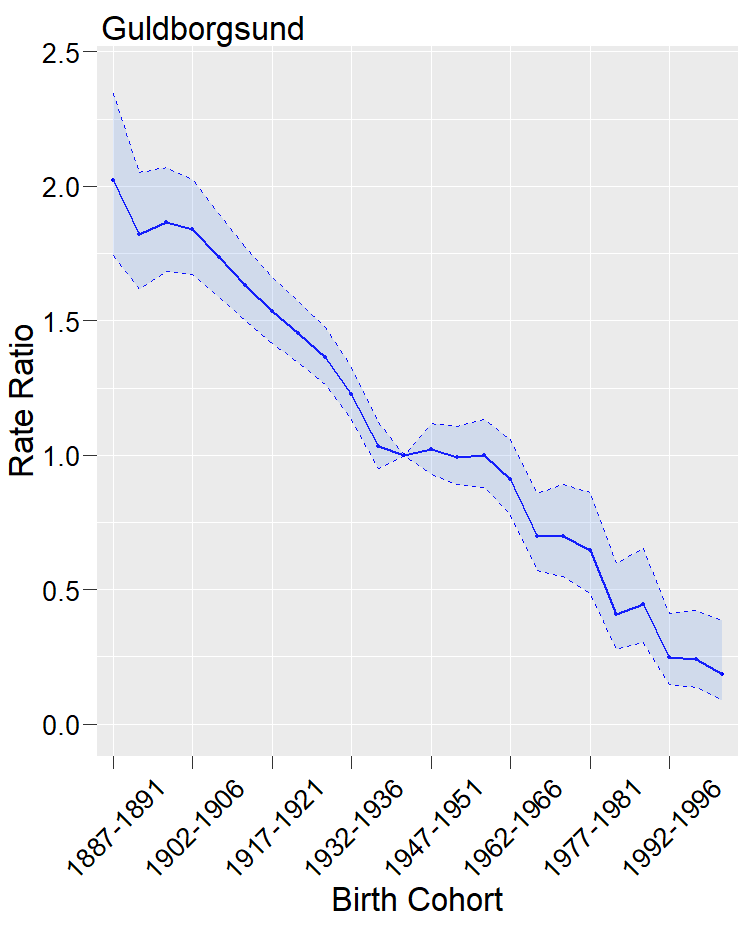

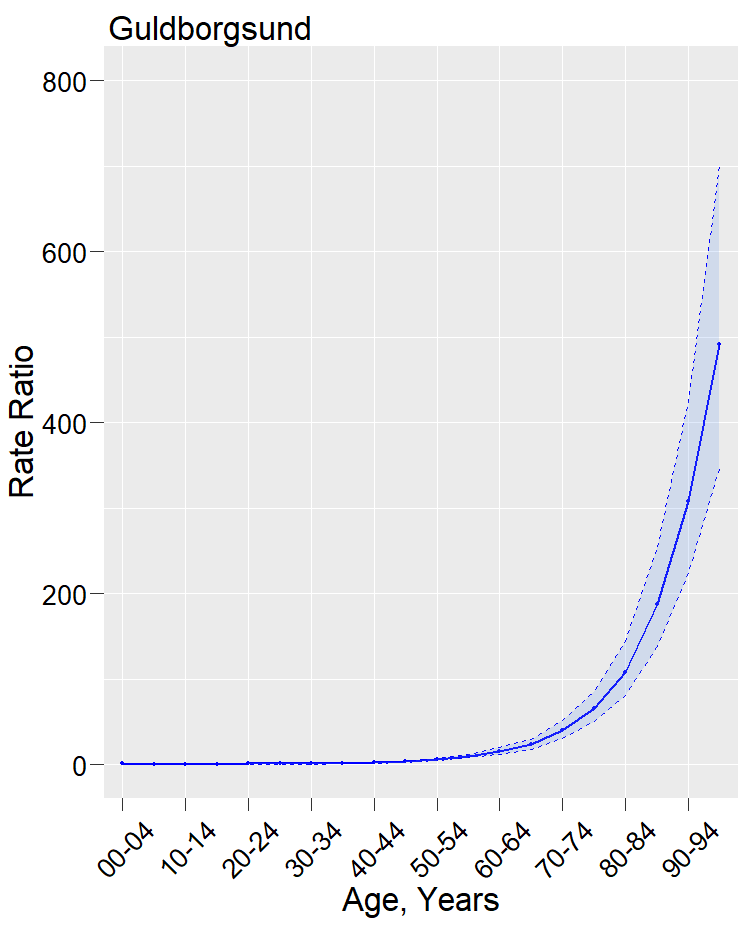


**Supplementary Figure 3** Age-period-cohort analysis as rate ratios for women in Denmark, Lolland and Guldborgsund municipalities. Estimates for year adjusted for age and birth cohort using 1974-77 as reference. Estimates for birth cohort adjusted for age and year using 1942-46 as reference. Estimates for age adjusted for year and birth cohort using 0-4 years as reference.


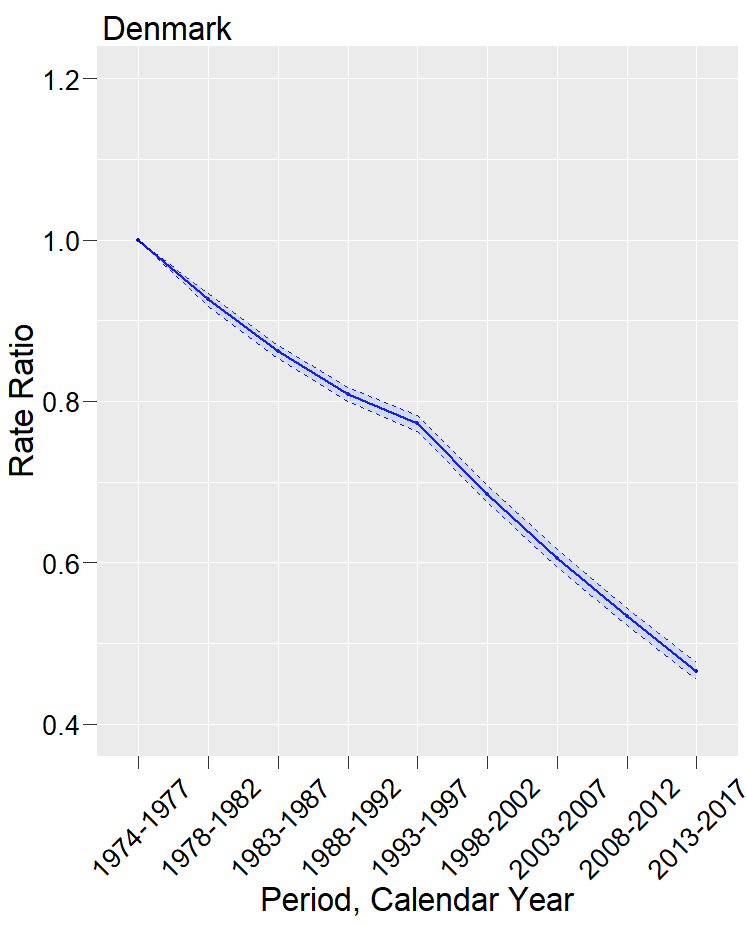

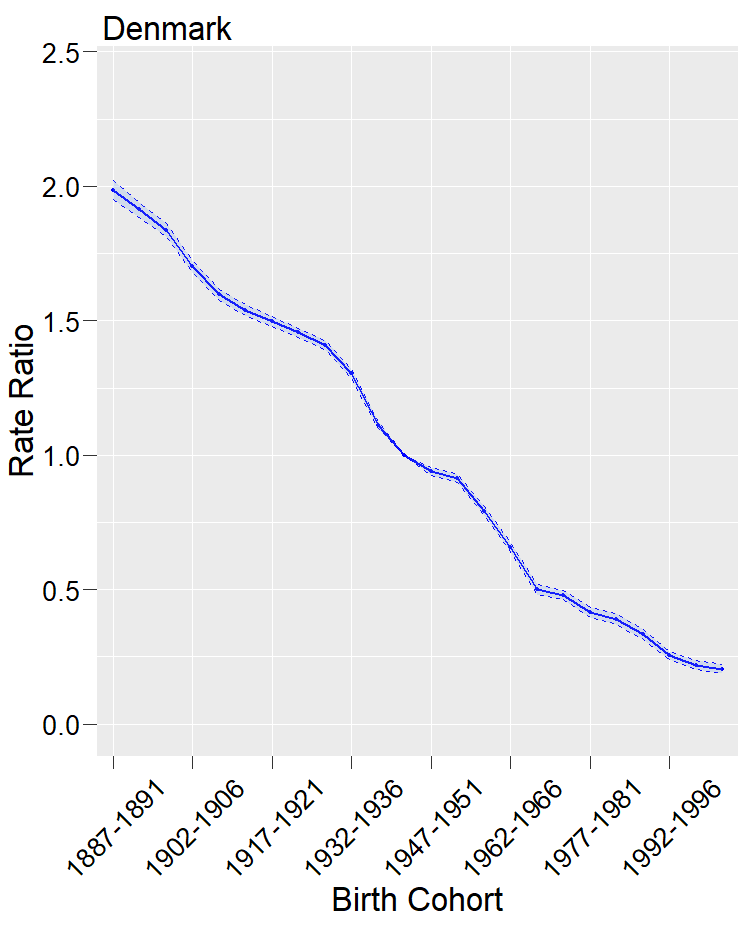

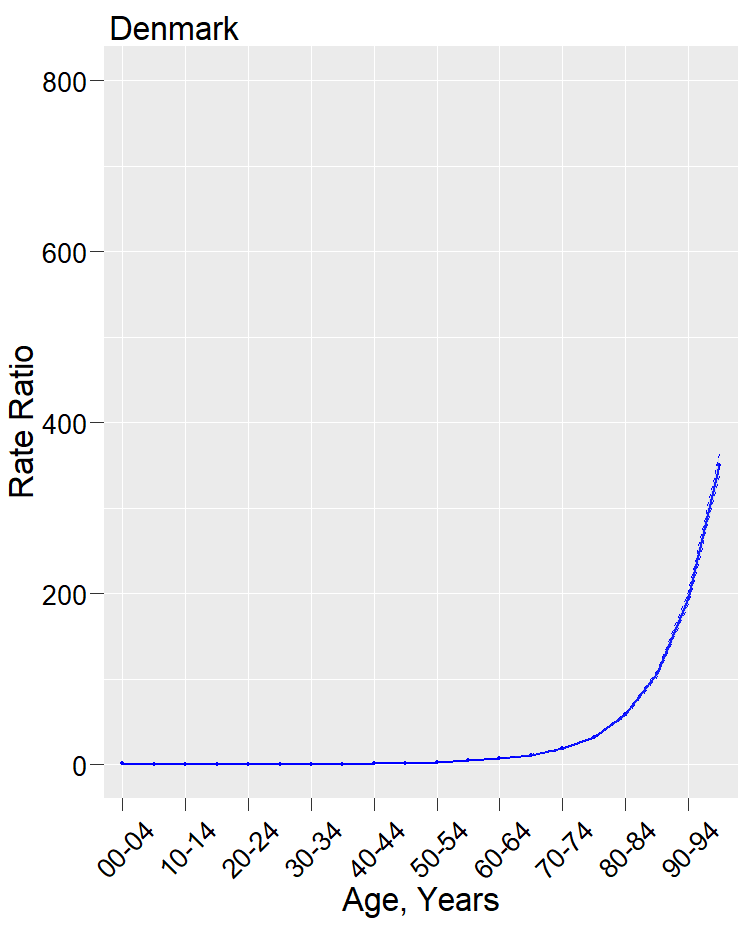

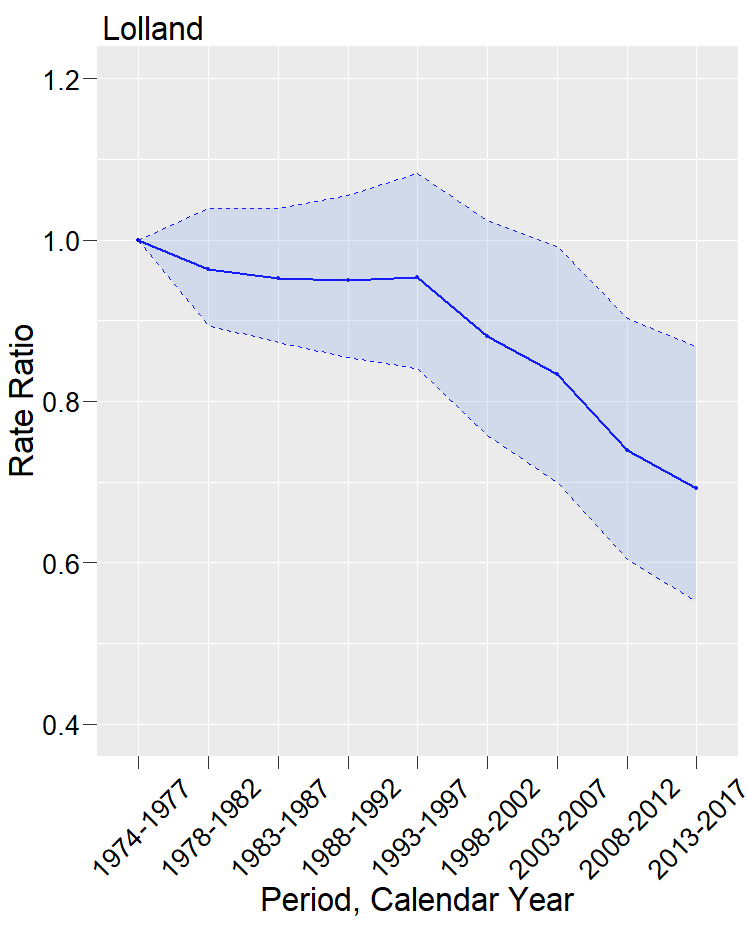

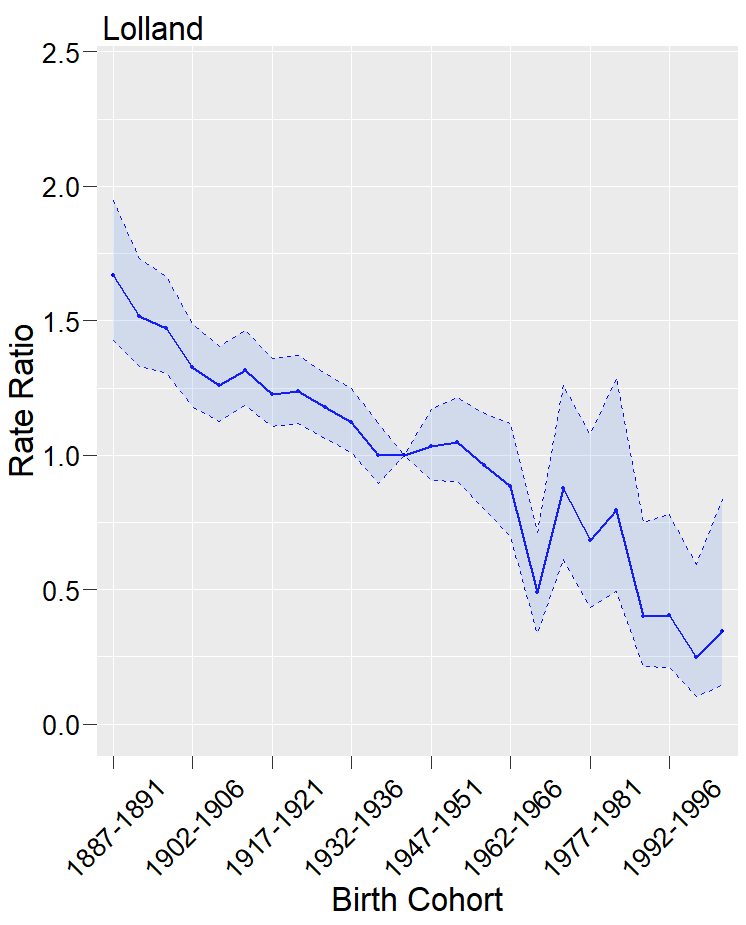

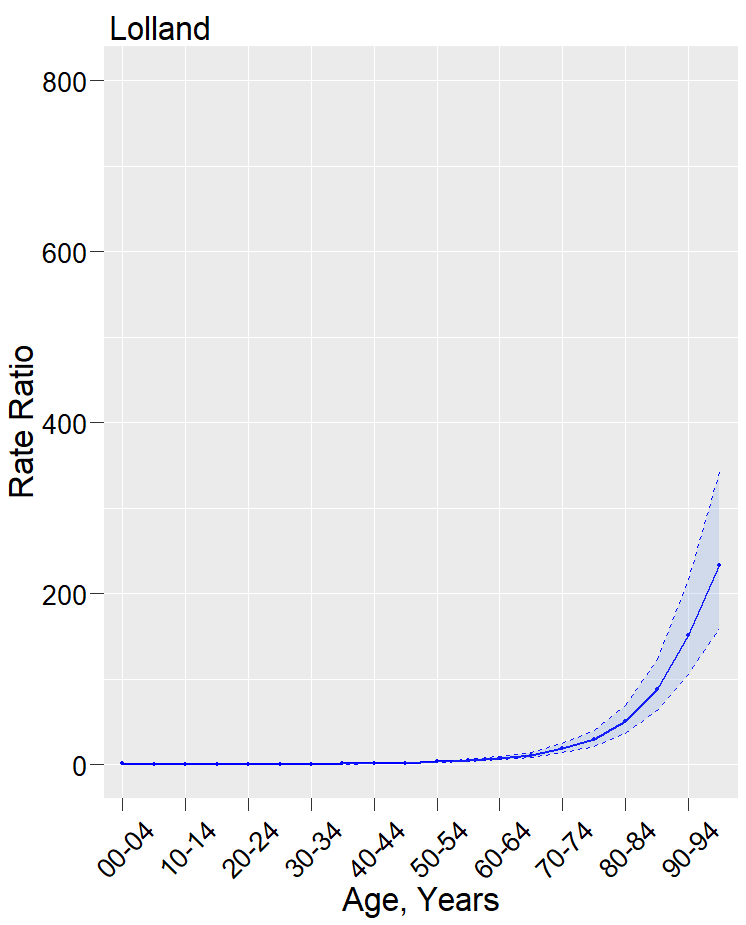

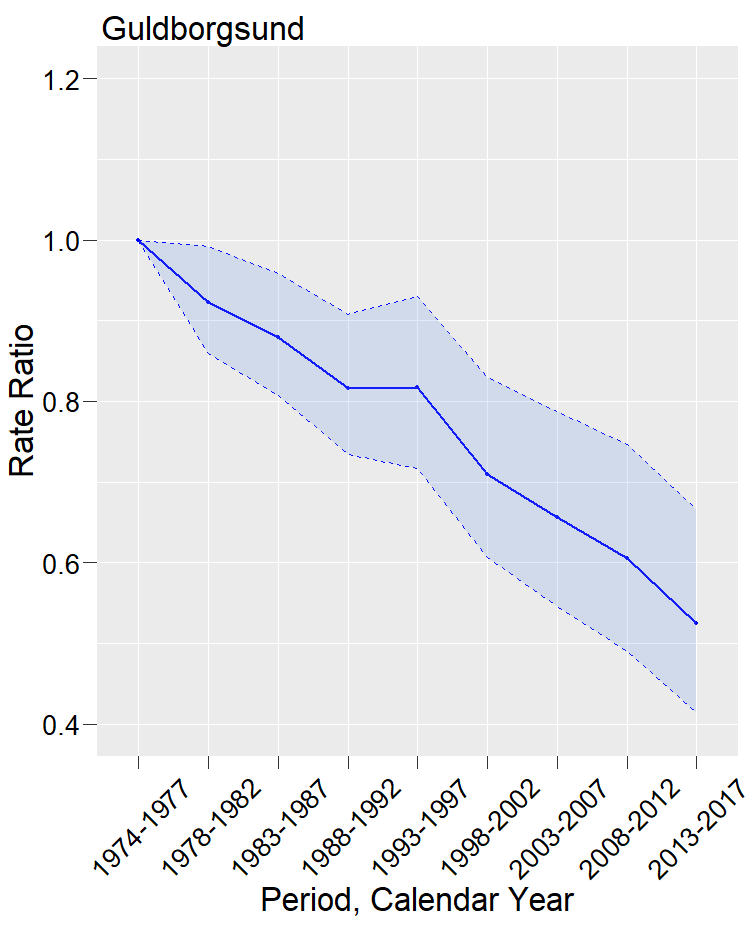

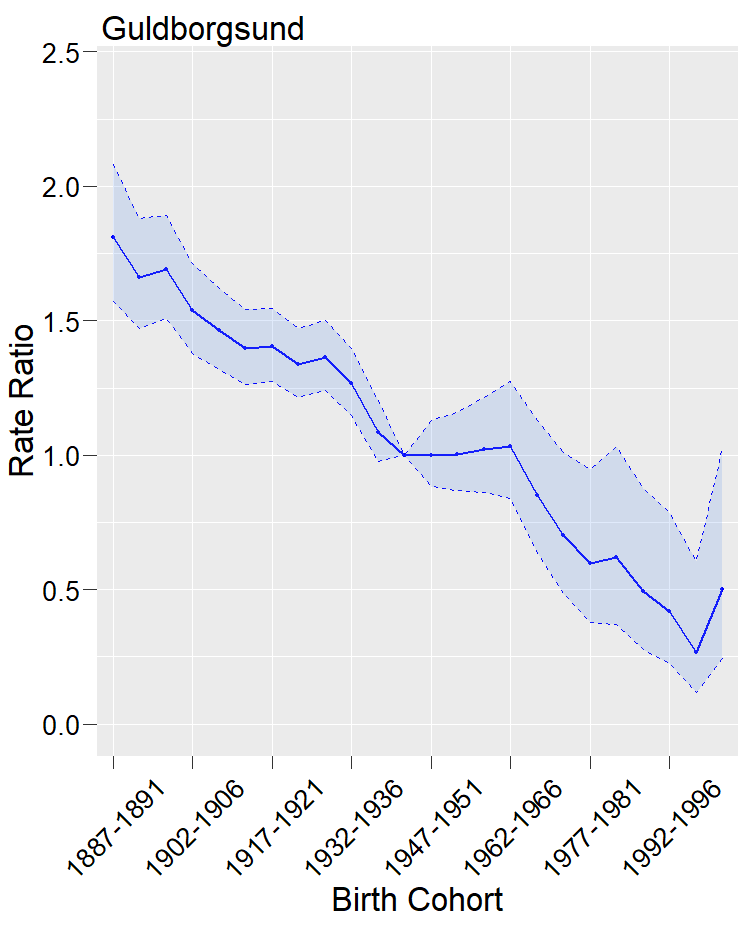

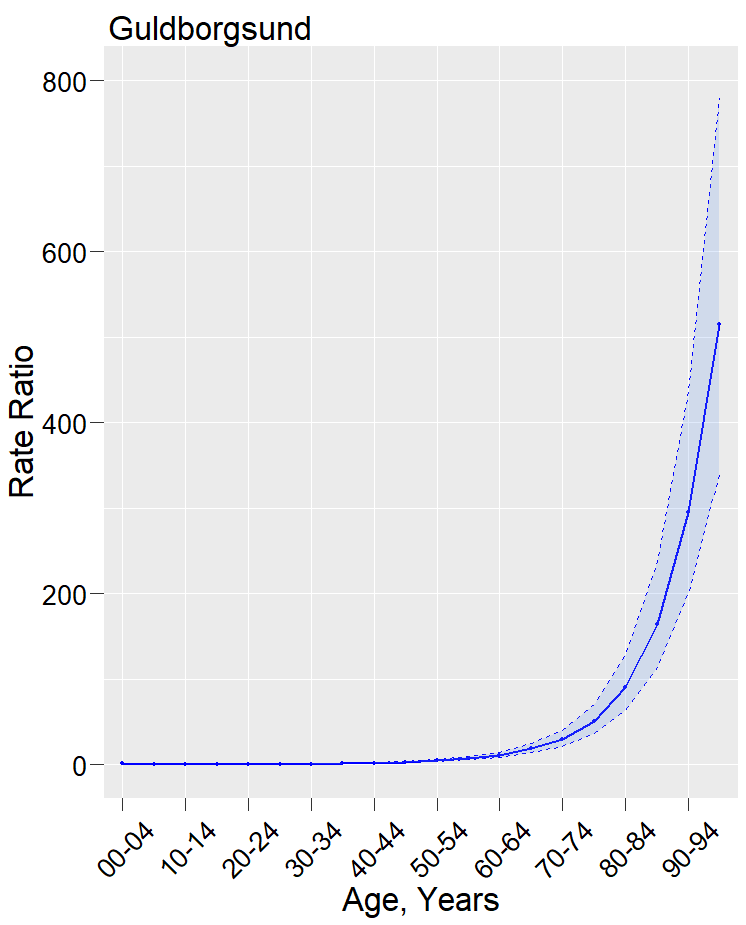


**Supplementary Figure 4** Standardised mortality ratio by cause of death for Lolland and Guldborgsund municipalities by sex (reference population = total population of Denmark).


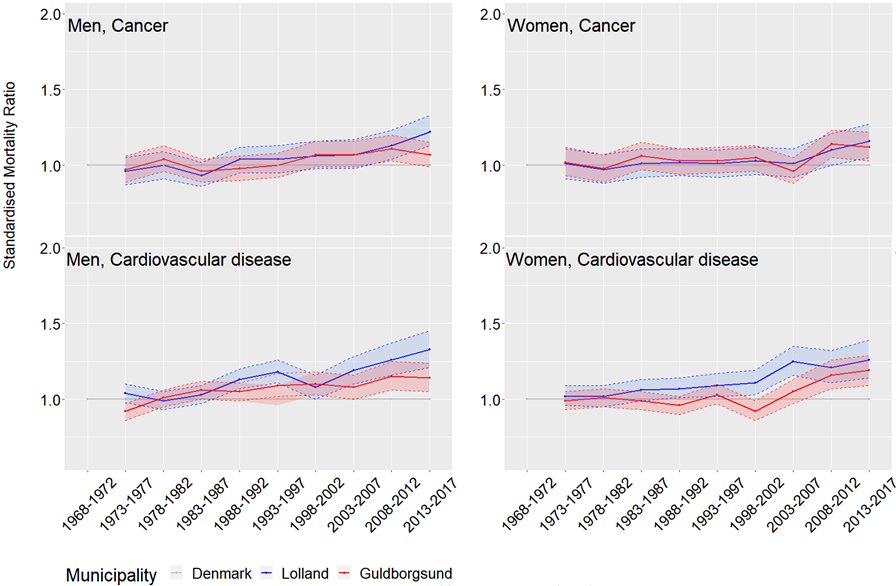


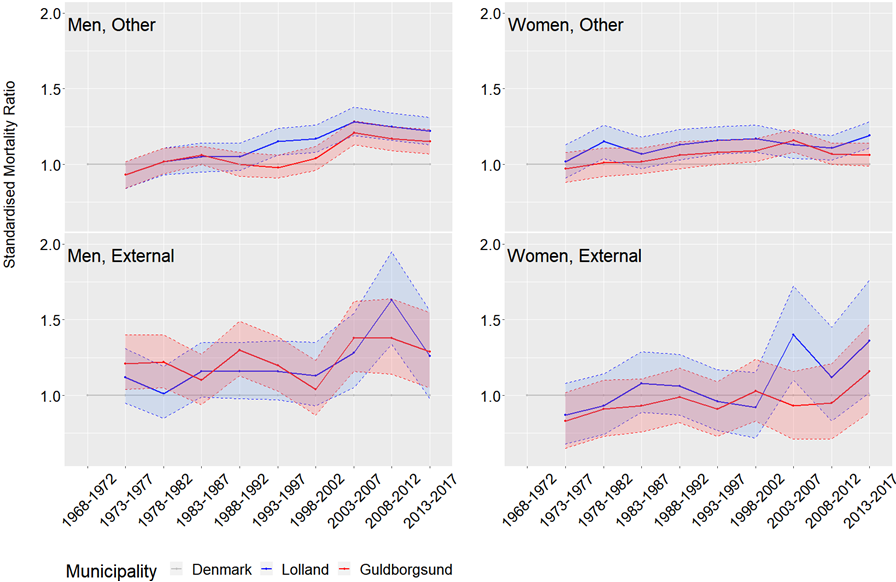


**Supplementary Figure 5** Life expectancy of geographical areas in Denmark for 10-year periods 1970-2019.
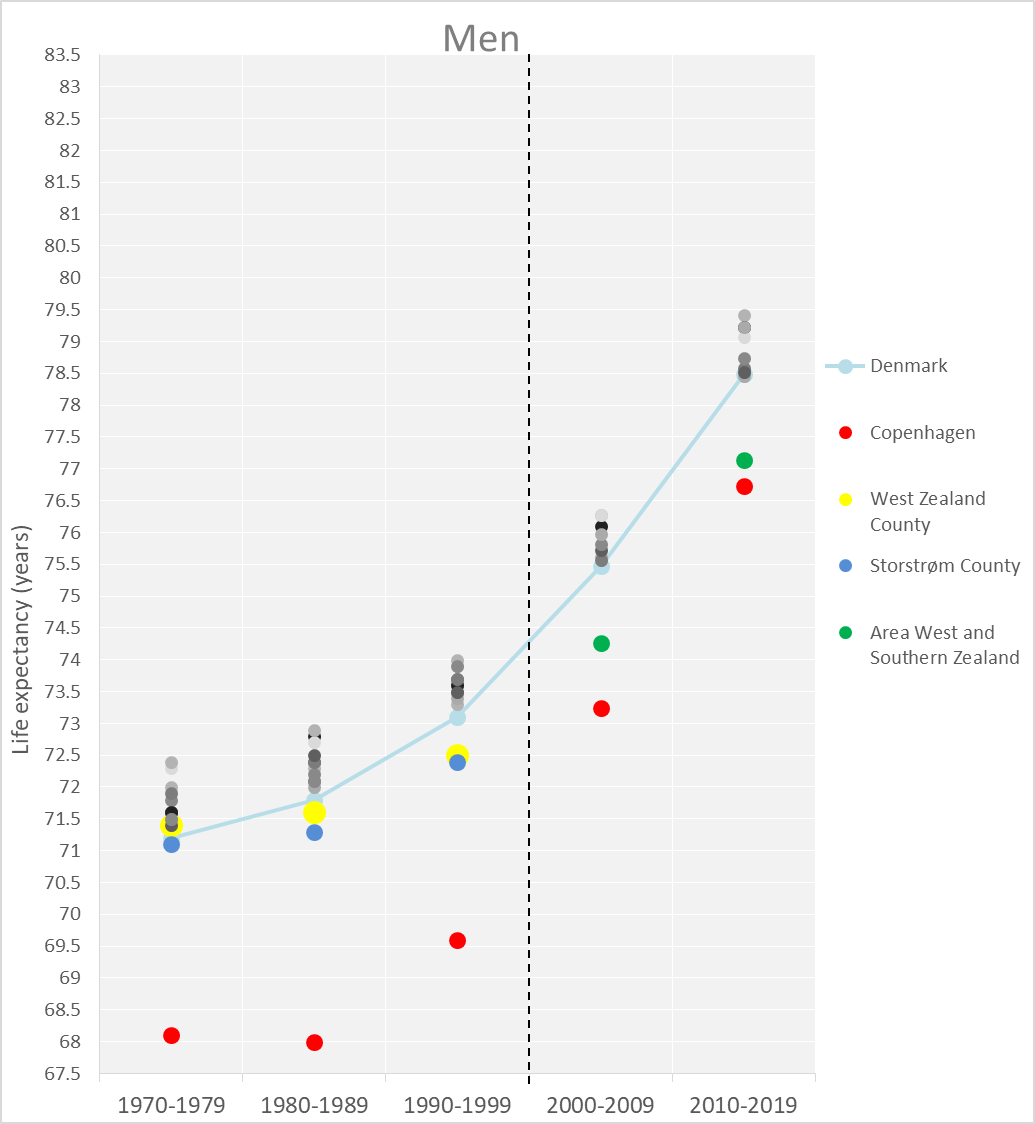


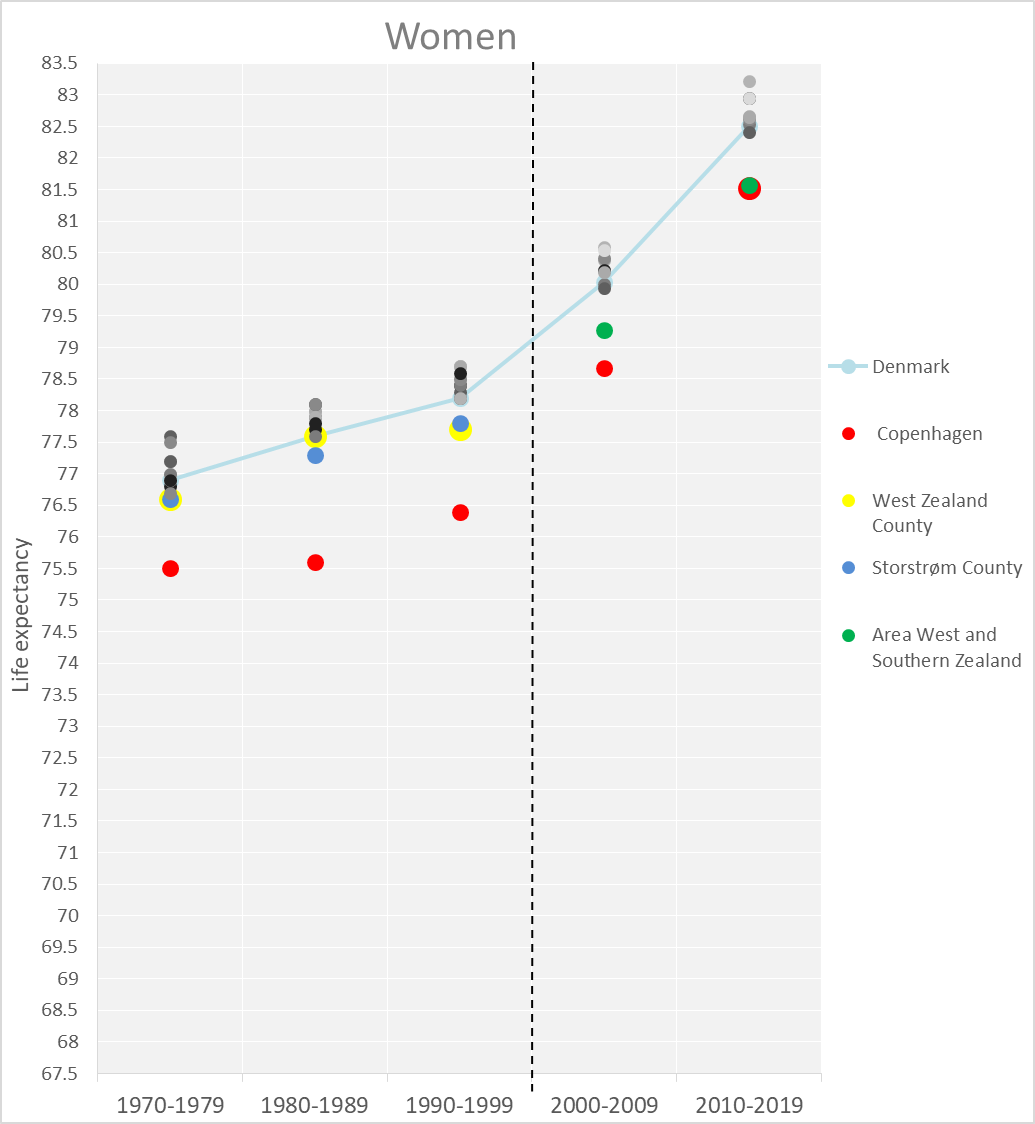


Note: 1970-1999: Old Danish counties [1]. 2000-2019: New Danish provinces [2]. Red: Copenhagen, from 2000 including the municipalities of Frederiksberg, Dragør and Tårnby. Blue: Old Storstrøm County. Yellow: Old West Zealand County. Green: New West and Southern Zealand. Grey/black: all other counties/provinces.

**References**

1. Juel K. Dødeligheden i Danmark gennem 100 år [Mortality in Denmark during 100 years]. Copenhagen; 2004.

2. Statistics Denmark. Life expectancy for new born babies by provinces and sex. StatBank Denmark. 2020.
